# A systematic review and meta-analysis of suicidality in autistic and possibly autistic people without co-occurring intellectual disability

**DOI:** 10.1186/s13229-023-00544-7

**Published:** 2023-03-15

**Authors:** Victoria Newell, Lucy Phillips, Chris Jones, Ellen Townsend, Caroline Richards, Sarah Cassidy

**Affiliations:** 1 School of Psychology, University of Nottingham, University Park, Nottingham, NG7 2RD UK; 2 School of Sport, Exercise and Health Sciences, Loughborough University, Epinal Way, Loughborough, LE11 3TU UK; 3 School of Psychology, University of Birmingham, 52 Pritchatts Road, Birmingham, B15 2TT UK

**Keywords:** Autism spectrum disorder, Autistic disorder, Asperger syndrome, Suicide, Suicidal ideation, Suicidal behaviour, Attempted suicide, Suicide, Self-injurious behaviour, Prevalence, Meta-analysis

## Abstract

**Background:**

Suicidality is highly prevalent in autistic people without co-occurring intellectual disabilities, and high autistic traits are found in adults who have attempted suicide. However, prevalence rates for both autistic and possibly autistic people have not been synthesised meta-analytically.

**Aims:**

To (1) calculate pooled prevalence estimates of suicidality in autistic people and possibly autistic people without co-occurring intellectual disability; (2) evaluate the influence of participant and study level characteristics on heterogeneity; and (3) determine the quality of evidence.

**Methods:**

Preferred Reporting Items for Systematic Reviews and Meta-Analysis guidelines were followed. PsycINFO, Embase, MEDLINE and Web of Science were systematically searched from 1992 to January 25, 2022. Empirical quantitative studies reporting prevalence of suicidal ideation, suicide plans, or suicide attempts and behaviours were considered for inclusion. Random effects models were used to estimate pooled prevalence of each suicidality outcome with 95% confidence intervals. Heterogeneity was explored using sensitivity and moderator analyses.

**Results:**

Data from 48,186 autistic and possibly autistic participants in 36 primary studies were meta-analysed. Pooled prevalence of suicidal ideation was 34.2% (95% CI 27.9–40.5), suicide plans 21.9% (13.4–30.4), and suicidal attempts and behaviours 24.3% (18.9–29.6). High levels of heterogeneity (*I*^2^ > 75) were observed in all three analyses. Estimates did not differ between autistic or possibly autistic samples. Geographical location (*p* = 0.005), transgender or gender non-conforming samples (*p* < 0.001) and type of report (*p* < 0.001) significantly moderated suicidal ideation, whereas age group (*p* = 0.001) and measure of suicidality (*p* = 0.001) significantly moderated suicide plans. There was a significant association between the proportion of male participants and prevalence of suicide plans, with a decrease in the proportion of males for every unit change of suicide plan prevalence (*p* = 0.013). No variables were found to moderate estimates of suicide attempts and behaviours.

**Conclusions:**

The results confirm suicidality is highly prevalent in both autistic and possibly autistic people without co-occurring intellectual disability and highlights potential moderators. Possibly autistic individuals require more attention in clinical and research considerations going forward to further understand and prevent suicide in both groups.

**Supplementary Information:**

The online version contains supplementary material available at 10.1186/s13229-023-00544-7.

## Introduction

People diagnosed with an Autism Spectrum Condition (ASC), henceforth autistic people,^1^ are characterised by differences in their social communication and interaction, sensory processing, focused interests, and preference for routine and familiarity [2]. Currently, it is estimated that 1.5% of the population in developed countries are autistic [3], with a male-to-female diagnostic ratio of approximately 3:1 [4]. Autism is highly heterogeneous, and it is well-established that autistic people often experience various physical health problems and psychiatric comorbidities [5]. Mental health problems in particular effect approximately 70–80% of autistic individuals across all age groups, with anxiety and depression being the most common and persistent of these [6–9].

In addition to high levels of mental health problems, autistic people are at a significantly increased risk of suicidality (suicidal ideation, suicide plans, suicide attempts, and death by suicide) compared to non-autistic people. An influential study of late diagnosed autistic adults found 66% had experienced suicidal ideation, which was nine times higher than the general population, and 35% had a suicide plan or had made a suicide attempt [10]. Moreover, a greater number of autistic adults are found to score above the psychiatric cut-off on measures of suicide risk compared to non-autistic adults [11, 12]. Large-scale population studies also report a four- and ninefold increase in death by suicide among autistic people compared to the general population [13, 14], and up to a sevenfold increase in suicide attempts [15], where this risk is the highest in autistic females and autistic people without co-occurring intellectual disability (ID) [13–16]. As suicide is a critical global health challenge and one of the leading causes of death worldwide [17], understanding this increased risk of suicidality in autistic people is essential for adequate risk assessment and preventative strategies.

Despite the concerning findings, the overall prevalence of suicidality in autistic people is highly variable across studies. Previous systematic reviews demonstrate that estimates range between 1 and 72% for suicidal ideation and 1 to 47% for suicide attempts in autistic individuals [18, 19]. Similarly, prevalence of suicidal ideation and suicidal behaviours in autistic samples under the age of 18 is found to range between 11 and 73% [20]. Possible explanations for this variation likely include a combination of diverse study and participant level characteristics, such as differences in sample size, recruitment from clinical or nonclinical settings [21, 22], and the way that suicidality is measured, reported, and defined [18]. Moreover age, gender and presence of co-occurring ID in autistic participants also differ greatly across samples as sources of variability [18]. Not only is it important to synthesise the current data on suicidality in autistic people, but also to understand the influence of which factors contribute the most significantly to these prevalence estimates.

An example of this variability includes the measures used to assess suicidality, which are inconsistent within the literature and have not been validated for use in autistic populations [11, 23]. Autistic people are found to interpret and respond to instruments designed for non-autistic people differently to what was intended by tool designers [24]. The Suicidal Behaviours Questionnaire—Autism Spectrum Conditions (SBQ-ASC) is the only tool that has recently been adapted for autistic populations but is therefore yet to be fully utilised in research [24]. Moreover, many studies, particularly with younger samples, use measures where items do not distinguish suicide attempts from self-injurious behaviour, such as the Child Behaviour Checklist [25] or the Paediatric Behaviour Scale [26]. While self-injurious behaviour is also highly prevalent in autistic people across all ages [27], the function of self-injurious behaviour and whether it is experienced with intent to end life is currently not well-enough understood to assess as commensurate to suicide attempts [28]. Additionally, measures that use informant-report may also lack sensitivity. Evidence of poor agreement is found between informant-report and an autistic person’s self-report on outcomes such as quality of life and mental health [29, 30]. If an autistic person’s experience of suicidality is not accurately and consistently captured, this may contribute to ranges in prevalence.

Regarding variability at the participant level, certain age groups of autistic people could contribute more to prevalence estimates of suicidality. Meta-analyses suggest suicidality varies with age in the general population, where adults (aged 18 +) demonstrate higher prevalence estimates than adolescents (aged 14–18), but older adults (aged 65 +) are at a lower risk compared to other age groups [31, 32]. If the developmental trajectory of suicidality follows a similar pattern in autistic people, we might expect age to account for some of the variability in prevalence estimates across studies. However, there is currently no research exploring this relationship in autistic people [28].

Gender may further explain some of the variance in prevalence. Males in the general population are 2.3 times more likely to die by suicide compared to females [17]; however, evidence suggests autistic females are at a higher risk of death by suicide and suicide attempts than autistic males [13, 15]. This may even be an underestimation, as autistic females frequently have their autism overlooked, misdiagnosed, or identified late [17], and can be inadvertently missed from relevant research as a result. Higher prevalence of suicidality is also found in autistic people who are transgender, and gender non-conforming compared to those who are cisgender (i.e. identify with sex assigned at birth) [33, 34]. Despite this, studies have only recently begun to acknowledge the joint impact of diverse gender identities and autism on mental health outcomes. Both female and transgender or gender non-conforming autistic people could therefore represent high risk groups that have a disproportionate influence on prevalence of suicidality.

Estimates could also vary depending on whether autistic people with and without co-occurring ID are included and analysed as separate groups within research. Some studies combine such groups into the same sample [e.g. 35–37], despite autistic people without co-occurring ID being at a greater risk of suicidality than those with co-occurring ID [13, 15, 18, 38, 39]. Prevalence may also be complicated by the frequent use of self-report for measures of suicidality, which are less accessible to individuals with co-occurring ID and may not provide an accurate representation of their internal experience [40]. For the purpose of this review, it is hoped that focusing on autistic people without co-occurring ID will reduce some of this ambiguity.

While the seriousness of suicidality in autistic people is evident, the reasons why this increased risk exists are still unclear and under-researched [28]. Similarly to within the general population, mental health problems, non-suicidal self-injury, unemployment and social isolation increase risk of suicide in autistic people; however these are significantly more prevalent [18, 41, 42]. Research also suggests there are risk factors for suicidality that are unique to autism, such as camouflaging (i.e. actively hiding autistic traits to be more accepted by non-autistic peers) and unmet support needs [41]. Given high rates of comorbid mental health problems [9, 43] and non-suicidal self-injury [44, 45] in autistic people, we might expect to see increased prevalence of suicidality in nonclinical samples of autistic people, who are less likely to be accessing relevant support [46]. Likewise, many autistic people find it difficult to initially obtain their diagnosis, whereby later age of diagnosis may also contribute to a lack of tangible support and increased suicidality [10]. There is currently no evidence for age of diagnosis as a risk factor for suicidality, but this has only been examined in autistic people diagnosed in adulthood so far [41]. The presence or absence of such risk factors within autistic samples should be considered in relation to the varying prevalence estimates of suicidality.

Finally, autism itself is thought to contribute to suicidality over and above other factors [41]. Possibly autistic people (i.e. individuals who score highly on measures of autistic traits but do not have an official ASC diagnosis) also appear to be at a higher risk of suicidality. Forty-one per cent (40.6%) of adults with a lifetime history of suicide attempt(s) were found to score above the clinical threshold for autistic traits [47]. Along with this, evidence of autism and elevated autistic traits were found in 10.7% of those who died by suicide in the UK [48]. Many individuals can go undiagnosed for various reasons, such as a lack of age-appropriate diagnostic services and tools to identify autistic females. This is particularly true for individuals who fit the profile of autism without co-occurring language delay or ID [49]. It is therefore important not to overlook these possibly autistic individuals when considering prevalence of suicidality.

To date, only two meta-analyses have examined suicidality in autistic people [21, 22]. One demonstrated approximately a threefold increase in the odds of suicidality (suicidal ideation, suicide attempts and suicide combined) in autistic people compared to non-autistic comparison groups, but did not examine suicidal ideation, suicide attempts or suicide as distinct outcomes [22]. The other meta-analysis only focused on studies with autistic youth, where pooled prevalence estimates were 25.2% for suicidal ideation, 8.3% for suicide attempts and 0.2% for death by suicide [21]. These meta-analyses provide useful findings, but do not address the prevalence of separate suicidality outcomes across the lifespan nor specifically for higher risk groups such as those without co-occurring ID and who are possibly autistic.

### Current aims

In summary, suicidality is worryingly common in autistic people, yet current prevalence estimates are highly varied, and the influence of participant  and study level characteristics on suicidality is unknown. Robust prevalence estimates of suicidality outcomes are therefore needed to identify the existing service needs of at-risk autistic individuals, and to inform evidence-based suicide prevention within this population. It is also necessary to explore the influence of participant and study level characteristics and evaluate the impact of these on the prevalence of suicidality outcomes in autistic individuals. To our knowledge this review is the first of its kind to examine studies of suicidality in both diagnosed autistic individuals and possibly autistic individuals, with a focus on those without co-occurring ID, across all age groups.

Thus, the aim of the current systematic review and meta-analysis is:

1. To synthesise prevalence estimates of suicidality in autistic people and possibly autistic people without co-occurring ID.
2. To evaluate the influence of participant (age, gender, autism or possible autism, presence of risk factors) and study level characteristics (study setting, geographical location, measurement of suicidality, type of report) on heterogeneity in prevalence estimates.
3. To determine the quality of evidence available.

## Methods

The review was conducted in line with guidelines for the Preferred Reporting for Items for Systematic Reviews and Meta-analysis (PRISMA) [50]. The protocol was pre-registered with PROSPERO before searches were undertaken (available at: https://www.crd.york.ac.uk/prospero/display_record.php?ID=CRD42021266451).

### Search strategy

A systematic literature search was carried out for papers published between 1992 and the search date, January 25, 2022. Four electronic databases (Embase, PsycINFO, MEDLINE and Web of Science) were reviewed using two search engines (PubMed and OVID) for studies examining the prevalence of suicidality in autistic and possibly autistic people without ID. Search terms (Table 1) were derived from recent systematic reviews [e.g. 11, 18], and were adapted to fit the specific search criteria of each database (see Additional file 1: Supplementary Materials 1 for full search terms and syntax). Reference lists of included primary studies and relevant prior systematic reviews or meta-analyses were also hand-searched for additional studies that may have been missed.

**Table 1:** Main search terms adapted for each electronic database

1. (ASC or ASD or Asperg* or Autis* or 'high#functioning' or 'pervasive developmental disorder' or PDD or HFA)
2. ('possib* autis*' or 'autis* trait*' or 'autis* phenotyp*' or 'undiagnosed autis*' or 'self-diagnos* autis*')
3. (suicid* or 'suicide plans' or 'suicide attempts' or 'attempted suicide' or parasuicide 'self-harm' or 'self-inj*')
4. #1 or #2
5. #3 and #4
6. Limit #5 to yr = ”1992—current”

*Wildcard search terms

### Selection strategy

Papers of empirical quantitative studies with extractable prevalence estimates were included. Searches were limited to studies available in the English language and those published after 1992. The cut-off date of 1992 was chosen to coincide with the official recognition of Asperger Syndrome by the International Classification of Diseases (ICD-10) [51], as subsequent research would be more likely to clearly differentiate autistic people without co-occurring ID.

Autistic participants were required to have a formal diagnosis of ASC in line with ICD (9 or 10) or Diagnostic and Statistical Manual of Mental Disorders (DSM-II, III-R, IV, IV-TR, V) diagnostic criteria (self-reported or confirmed within the study). Possibly autistic participants were required to self-report suspected autism (not yet diagnosed) and/or screen positive for elevated autistic traits on a relevant measure (e.g. Autism Quotient). Data for autistic and possibly autistic participants had to be provided separately from any additional groups. Studies were excluded if any proportion of autistic or possibly autistic participants were specified to have co-occurring ID or an IQ below 70, or when data for participants without co-occurring ID was not provided or analysed separately to those with co-occurring ID. Where studies did not specify IQ or confirm ID status, but it could be inferred through other means (e.g. self-report, level of education), this was taken as an acceptable indicator.

Studies were included if suicidality was clinically defined based on the ICD-10 or DSM-5; encompassing suicidal ideation (or thoughts), plans, and attempts or behaviours. Studies of self-harm or self-injury without suicidal intent (e.g. non-suicidal self-injury), where suicidal intent could not be determined, or where measurement items did not distinguish suicidality outcomes from self-harm or self-injury, were not included. This is in line with a dichotomous conceptualisation of self-harm consistent with previous autism research where non-suicidal self-injury and suicidality are generally examined as separate constructs [28, 52]. Full eligibility criteria are described in Table 2.

**Table 2:** Eligibility criteria used for study selection during title, abstract and full-text screening

Inclusion criteria	Exclusion criteria
Participants with a formal diagnosis of ASC Participants who are possibly autistic (but undiagnosed) Data for autistic and/or possibly autistic participants provided separately to any additional groups Prevalence estimates of suicidality reported using ICD or DSM clinical definition Empirical quantitative studies, following cross-sectional, longitudinal, cohort or case–control designs Published from 1992 to present day	Autistic or possibly autistic participants with co-occurring intellectual disability Prevalence estimates only provided for self-harm or self-injury without suicidal intent, or where measurement items do not distinguish these from suicidality Conference abstracts, conference papers, review articles, editorials or book chapters Grey literature (e.g. theses) Empirical qualitative studies, or other systematic reviews and meta-analyses Not published and/or available in English

### Selection process

Electronic searches of the databases identified 4560 potentially eligible studies. After the removal of duplicates, 1995 studies were then screened for eligibility using the criteria at title and abstract, and then 359 at full text by the first author (VN). Those that did not meet the selection criteria were excluded. If there was uncertainty regarding an article at the title and abstract screen, it was put forward for a full-text screen.

To eliminate the risk of researcher bias, 25% of papers at both stages were checked by an independent reviewer (LP). Inter-rater reliability was calculated using percentage agreement and Prevalence- And Bias-Adjusted Kappa (PABAK) [53], where strength of agreement was determined by PABAK as poor (< 0.20), fair (0.21–0.40), moderate (0.41–0.60), good (0.61–0.81) or very good (0.81–1) [54]. Agreement was fair for the title and abstract screen (66.18%, PABAK = 0.23), and moderate for the full-text screen (86.17%, PABAK = 0.55). All discrepancies were discussed to reach a consensus, and where this could not be resolved, the opinion of a third reviewer was sought (SC, CR).

### Data extraction and synthesis

From the studies eligible for the review, data of interest was manually extracted by the first author (VN). This included:

(i) Citation level data—author name(s), year of publication, and geographical location.
(ii) Participant level data—whether participants were autistic (with an ASC diagnosis) or possibly autistic (e.g. scoring above threshold on a measure of autistic traits); total number of participants; absolute number of participants with suicidality outcomes; age; gender; whether sample was transgender or gender non-conforming; sample setting; and where available, comorbidities, age of diagnosis, proportion of participants in employment (full-time, part-time, or volunteering), and presence of non-suicidal self-injury.
(iii) Study level data—study design; ascertainment of autism or possible autism; measure of suicidality; type of report used in suicidality measure; and observation period of suicidality assessment.

Prevalence was classified into outcomes of suicidal ideation, suicide plans, or suicide attempts and behaviours. Suicide attempts and behaviours covered both suicide attempts and estimates of suicidal behaviour where it was unclear whether this was a suicide attempt per se but was still assumed to have had suicidal intent. Prevalence for each suicidality outcome was established from the absolute number of autistic or possibly autistic individuals experiencing suicidality and the total number of autistic or possibly autistic participants.

Some studies provided prevalence for more than one suicidality outcome, meaning a single study could contribute to multiple pooled prevalence estimates in the review. If no absolute number of events could be obtained, authors were contacted to provide the information, or this was calculated from the related proportion and total number of participants. In circumstances where it was clear that multiple studies had used the same sample or dataset, these were evaluated and the one which was most relevant to the objectives of the review was included in the quantitative synthesis.

Age was stratified into two subgroups based on previous research [22]. If mean age of participants at enrolment was younger than 20 years, the age group was classified as youth, and where mean age was 20 years-old and above, the age group was classified as adult. Where mean age was not available, the median or midpoint of the given age range was used instead. Study setting was defined as clinical if participants were recruited from a clinical population or setting (e.g. outpatient clinics, emergency departments), and nonclinical if participants were obtained from a community or population sample, databases, or other. Subgroups were classed as transgender or gender non-conforming when all participants in that sample or group did not identify with or were questioning their sex assigned at birth. The proportion of males was reported more frequently compared to females, so this was used as the indicator of gender.

### Quality assessment

Quality of studies included in the final synthesis were assessed using an adapted Newcastle–Ottawa scale (NOS) [55] based on versions used in previous research [21, 56] (see Additional file 1: Supplementary Materials 2). The NOS is widely used to evaluate the methodological quality of observational studies [57] as it can be easily adapted to be study specific, is straightforward to administer, and provides a continuous score that is consistent across study designs.

We assessed the following criteria: (1) selection, (2) comparability, and (3) outcome for cross-sectional and cohort studies; and (1) selection, (2) comparability and (3) exposure for case–control studies. Items were adapted in line with the specific aims of the review. Sample representativeness was determined from both the sampling method and using the 3:1 male-to-female autism diagnostic ratio (i.e. at least 25% of participants were required to have either been assigned female at birth or identify as female) identified in previous research [4]. Sample size was deemed justified and satisfactory where there was statistical evidence of adequate power reported in the paper, or where the sample size would be considered large enough (*n* > 1000) to account for heterogeneity between and within autistic and possibly autistic individuals [58].

Primary studies were given an overall score of 0 to 9 and a rating of high (0–3), unclear (4–6) or low risk of bias (7–9) based on this score. VN assessed the quality of all studies, and 50% of these were independently checked by LP. Agreement was moderate (80%, PABAK = 0.60). All discrepancies were discussed, and a joint consensus was reached.

### Statistical analysis

Individual pooled prevalence estimates were generated for suicidal ideation, suicide plans, and suicide attempts and behaviours in autistic and possibly autistic people without co-occurring ID. Statistical power was not adequate to conduct meta-analyses for each suicidality outcome in just the possibly autistic group (*k* ≥ 10) [59]. Therefore, autistic and possibly autistic groups were meta-analysed as one but explored as a potential moderator using subgroup analyses. Given substantial heterogeneity in the extracted prevalence estimates, the random effects model was chosen as the most appropriate method of meta-analysis. Random effects models assume that both variability in sampling error and differences in study level characteristics account for heterogeneity between studies [60]. The decision to use random effects was supported with Quantile–Quantile plots as an indicator of primary study effects relative to that of an expected normal distribution.

Between studies variance (tau^2^) was calculated with the restricted maximum-likelihood estimator (REML), which is considered more robust to non-normal distributions of effect than the more traditional DerSimonian Laird estimate [61]. Level of heterogeneity within studies was established with Higgins *I*^2^, where a value above 75% suggested high heterogeneity [60] and significance was quantified using Cochran’s Q statistic. Prediction intervals were provided alongside pooled prevalence estimates and confidence intervals. While a 95% confidence interval indicates where, in 95% of cases, the average prevalence estimate will fall; the 95% prediction interval indicates where, in 95% of cases, the true prevalence estimate of a new study will fall [62]. When heterogeneity is high, prediction intervals are expected to be wider than confidence intervals to account for between study variability and provide a more conservative way to incorporate uncertainty in analyses [63].

The impact of influential and discrepant studies on the overall meta-analytic effect was explored using Baujat plots and “leave-one-out” sensitivity analyses. The Baujat plot shows the contribution of each study to the overall heterogeneity statistic on the x-axis and its influence on the pooled effect size on the y-axis [64]. Higher values on the x-axis reflect increasing heterogeneity associated with omission of a study, whereas higher values on the y-axis indicate greater change in the overall effect associated with a studies omission. Therefore, those in the upper right corner may be particularly influential [64]. This influence is further determined by a “leave-one-out” sensitivity analysis, where a random effects model is calculated with each of the primary studies removed in turn. Based on a rule of thumb used in previous research [27], if omission of influential primary studies resulted in an effect that lay outside the 95% confidence interval for the complete meta-analysis, it was deemed to have a disproportionate influence on prevalence and excluded from subsequent analyses.

Visual inspection of funnel plots were used to detect publication bias in each meta-analysis. This was also quantitatively informed with Egger’s regression test of asymmetry when at least 10 estimates were included [65]. For the funnel plot, effect estimates were plotted on the horizontal axis and the measure of study size on the vertical axis. In the absence of publication bias, the plot should resemble a symmetrical funnel-shaped distribution where lower precision studies scatter widely on both sides of the average, with the spread narrowing among larger studies, and those of highest precision at the top [66].

An absence of studies in the area of the funnel plot associated with small or non-significant effects sizes in smaller studies indicated publication bias. In this case, a trim and fill procedure was undertaken to identify and correct for funnel plot asymmetry by estimating the number of unpublished studies and imputing these missing values to provide a pooled prevalence estimate adjusted for publication bias [67]. This was then compared with the uncorrected random effects model. Orwin’s Fail-safe N [68] also calculated the number of studies with null results that would have to be added to reduce the observed meta-analytic effect to that of the general population [31, 69]. If N is large, the effect can be considered robust to publication bias.

Further moderator analyses were conducted to explore heterogeneity related to participant and study level covariates. These were determined post hoc based on the available data and were only performed when a minimum of 10 estimates were available to ensure adequate statistical power [59, 70]. For each meta-analysis that met this requirement, prevalence estimates and associated heterogeneity measures were calculated and compared for the following categorical variables: group (autistic vs possibly autistic), age (youth vs adult), (Asia vs Europe vs North America vs Oceania), sample setting (clinical vs nonclinical), transgender or gender non-conforming (yes vs no), and type of report for suicidality measure (self vs informant vs observational). Meta-regression analyses were also used to assess the relationship between continuous moderators and each outcome. Insufficient data were available for age of diagnosis, proportion employed, and proportion reporting non-suicidal self-injury; therefore, it was only possible to explore the continuous variables for proportion of male participants (%) and year of publication. Each potential moderator was assessed in separate univariate analyses (including a different covariate), and the corresponding results were interpreted.

Primary studies were included regardless of their quality score. Sensitivity analyses were conducted to ascertain the impact on the pooled prevalence estimate of each random effects model using a meta-regression of the adapted NOS overall score, and a subgroup analysis of risk of bias (low risk vs any (unclear and high) risk).

## Results

**Fig. 1:**
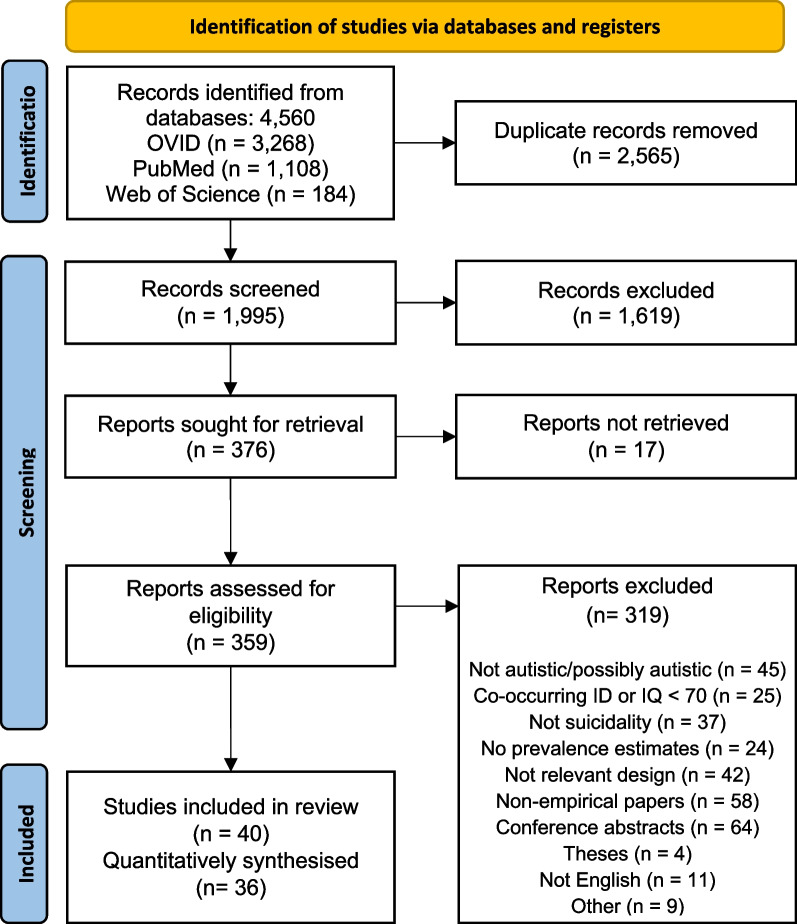
PRISMA flow diagram for the selection of articles

The searched databases yielded 4560 potentially eligible studies published between 1992 and January 25, 2022. Of these, 2565 studies were identified as duplicates through referencing software or hand searching, then removed. The remaining 1995 studies were screened at title and abstract using the criteria in Table 2. Seventeen studies were not accessible to the authors and could not be screened further. Full-text screening was conducted for 359 studies, 319 of which were excluded for the following reasons: 45 did not meet the criteria for the autistic or possibly autistic sample; 25 contained participants with ID or an IQ below 70; 37 did not meet the criteria for suicidality; 24 did not report prevalence estimates for suicidality outcomes; 42 did not meet the criteria for study design; 58 were not empirical papers; 64 were conference abstracts; 4 were theses or dissertations; 11 were not available in the English Language; and 9 were excluded for other reasons, such as using the same sample or data from another study. The full selection process according to PRISMA guidelines is depicted in Fig. 1.

### Study characteristics

Forty primary studies with extractable prevalence rates were included (see Table 3). This represented 48,692 autistic and possibly autistic participants without co-occurring ID (autistic *n* = 46,875; possibly autistic *n* = 1817). Over two-thirds of studies included only autistic participants (*k* = 31; 77.5%), 10% included only possibly autistic participants (*k* = 4), and 12.5% included both groups (*k* = 5). Twenty-three primary studies included autistic adults (age ≥ 20 years; 57.5%) and 17 included autistic youth (age < 20 years; 42.5%). Of primary studies which gave a mean age, this ranged from 10.1 (SD = 2.7) to 42.3 (SD = 13.9). Thirteen studies contained a predominately male sample (32.5%), where the proportion of males made up 75% or more of the autistic or possibly autistic participants.

**Table 3:** Participant, study characteristics and outcome data for studies reporting the prevalence of suicidality in autistic and possibly autistic people

Study			Autism		Participant characteristics	Measures		Prevalence
Authors	Country	Design	Group	Comparator	Sex, Age, Age of diagnosis, IQ, Comorbidity, Employment	Autism and Possible Autism	Suicidality measure, type of report, timeframe, specific items/questions	Suicidality
Anderson et al. (2020)	Australia	Cross-sectional	Nonclinical 102 Autistic	-	54.6% Male Age range 18–25 + Age diagnosed: 0–12 years (40%), 13–17 years (21%), 18 + years (37%), Unknown (2%) Q unknown (*university students*) Comorbidity: Depression (40%), Anxiety (68%), ADHD (31%), SH (16%)	Self-identified formal diagnosis	Unstandardised (self-repor*t*) Unspecified timeframe *Optional questions "During the time I have been a university student… I have never thought about suicide/ I have thought about suicide, but I would not act on those thoughts/ I have made plans to suicide, but I did not act on those plans/ I have attempted suicide"*	SI: 37 (48%) SP: 9 (12%) SAB: 1 (1%)
Arwert & Sizoo (2020)	The Netherlands	Cross-sectional	Clinical 75 Autistic	-	61.1% Male Age range 19–64 (M = 35.9, SD = 12.96) IQ threshold > 70 Comorbidity: Measured symptoms of Depression Employed: paid (42.7%), voluntary (25.3%)	Formal diagnosis	BSS (self-report) SI: Past 2 weeks SA: Lifetime	SI: 47 (65.3%) SAB: 21 (29.2%)
Bal et al. (2021)	Korea	Cross-sectional	Nonclinical *86 Autistic (KP ASC)*^*1*^ 1292 Possibly autistic (864 KP/ 223 CHEER/ 205 Sooncheon)	3 population-based samples (14,423 KP/ 3702 CHEER/ 4837 Sooncheon)	*Autistic* *80.2% Male* *Age range 7–12 (M* = *8.88, SD 1.60)* *IQ (M* = *93.84, SD* = *25.3; WISC-III), 17.4% had IQ* < *70* *Comorbidity: Anxiety (38.4%), Hyperactivity (55.8%)* Possibly Autistic 62.5% Male KP (M = 9.43, SD = 1.69); CHEER (M = 10.91, SD = 0.98); Sooncheon (M = 8.82, SD = 1.53) IQ unknown *(assumed to have intellectual function in the average range)* Comorbidity: Anxiety (KP 38.6%; CHEER 29%; Sooncheon 38.6%), Hyperactivity (KP 55.3%; CHEER 57.9%; Sooncheon 64.9%)	ADI-R ADOS ASSQ DSM-V	BASC-2 PRS-C (informant-report) Past several months *Item 92 "I want to die" or "I wish I were dead" and item 138 "I want to kill myself"*	Autistic *KP ASC SI: 12 (14%)* Possibly KP SI: 171 (19.8%) CHEER SI: 61 (27.4%) Sooncheon SI: 34 (16.6%)
Balfe & Tantam (2010)	UK	Cross-sectional	Nonclinical 42 Autistic	-	88.1% Male Age range 13–64 (M = 26.21, SD = 11.9) IQ threshold ≥ 70 Comorbidity: Depression (35%), Anxiety (51%), Alcohol problems (15%), Drug problems (10%) Employed: paid (28%), voluntary (25.3%)	ADI-R Documented proof	Unstandardised (self-report) Unspecified timeframe *"Have you ever thought about killing yourself?" and "Have you ever tried to kill yourself?"*	SI: 17 (40%) SAB: 6 (15%)
Bemmouna et al. (2021)	France	Intervention	Clinical 7 Autistic	-	57% Male (n = 3) Age range 19–56 (M = 27.71, SD = 13.34) IQ threshold > 80 (M = 108.8, SD = 25.69; WAIS-IV) Comorbidity: BPD (14%), ADHD (29%), SH (57%) Employed: 14%	ADI-R ADOS-2	Unstandardised pre-inclusion interview questions (self-report) SI: Past year SA: Lifetime *"Do you have any history of suicide attempts?/ In difficult situations, some people may have suicidal ideation. Does this apply to you?”*	SI: 5 (71%) SAB: 5 (71%)
Cassidy et al. (2021)	UK	Cross-sectional	Nonclinical 308 Autistic (formal diagnosis) 113 Possibly autistic (suspected diagnosis)	268 Non-autistic	Autistic 27% Male Age (M = 39.71, SD = 13.34) IQ unknown (*without ID specified*) Comorbidity: Depression (50%), Anxiety (59.74%), PTSD (17.86%), OCD (9.42%), Bipolar Disorder (4.55%), Anorexia (3.9%), Bulimia (1.62%), Personality disorder (6.49%), ADHD (13.64%), lifetime NSSI (62.7%) Employed: full-time (30.19%), part-time (21.43%), voluntary (10.71%) Possibly Autistic 29.2% Male Age (M = 40.34, SD = 13.55) IQ unknown (*without ID specified*) Comorbidity: Depression (39.82%), Anxiety (44.25%), PTSD (13.27%), OCD (2.65%), Bipolar Disorder (2.65%), Anorexia (0.88%), Bulimia (0.88%), Personality disorder (7.96%), ADHD (7.96%), lifetime NSSI (46.4%) Employed: full-time (41.49%), part-time (16.81%), voluntary (7.07%)	AQ Self-identified formal and suspected diagnosis	SBQ-R (self-report) Lifetime	Autistic SI: 86 (27.9%) SP: 90 (29.2%) SAB: 113 (36.7%) Possibly Autistic SI: 53 (46.9%) SP: 24 (21.2%) SAB: 26 (23%)
Cassidy et al. (2018)	UK	Cross-sectional	Nonclinical 164 Autistic (65 Male/ 99 Female)	169 General Population (54 Male/ 115 Female)	39.6% Male IQ unknown (without *ID specified*) Comorbidity: Depression (79.8%), Anxiety (71.3%), OCD (14.6%), Bipolar (4.9%), Anorexia (5.5%), Bulimia (1.2%), Schizophrenia (3.7%), Personality Disorder (14%), ADHD (6.7%), NSSI (64.6%) Male Age range 20–60 (M = 41.52, SD = 11.73) Age at diagnosis (M = 34.55, SD = 14.75) 46.2% Employed FemaleAge range 20–60 (M = 38.89, SD = 10.47) Age at diagnosis M = 35.06, SD = 11.83) 51.5% Employed	AQ-S Self-identified formal diagnosis	Unstandardised patient screening questionnaire (self-report) Lifetime *“Have you ever felt suicidal? If yes, have you ever planned or attempted suicide?”*	*SBQ-R* ≥ *8: 118 (72%)*^*2*^ SAB: 63 (38.4%)
Cassidy et al. (2014)	UK	Cross-sectional	Nonclinical 374 Autistic	-	68% Male Age range 17–67 Age at diagnosis (M = 31.5, SD = 10.9) IQ unknown (*diagnosed AS*) Comorbidity: Depression (32%), OCD (1.1%), Eating Disorder (0.5%), ADHD (1.1%)	AAA AQ Clinical judgement	SBQ-ASC (self-report) Lifetime	SI: 243 (66%) *SP/SA: 127 (35%)*^*2*^
Chang et al. (2021)	Taiwan	Cohort	Clinical & TGNC 88 Autistic (24 GD/ 64 No GD)	42 age + sex matched TD	89.8% AMAB Age range 17–24.5 (*at follow up:* M = 20.4, SD = 2.0) IQ threshold ≥ 70 (M = 97.40, SD = 20.47; WISC-III, WAIS-III) Comorbidity excluded: Any major psychiatric disorders, SH (13.6%)	ADI-R (Chinese) DSM-IV-TR SRS	ASRI-4 (Chinese; self-report) Lifetime *Item 41 “I think about death or suicide” and item 50 “Have you ever attempted suicide”*	GD SI: 11 (45.9%) No GD SI: 18 (28.1%) GD SA: 2 (8.3%) No GD SA: 5 (7.8%)
Chaplin et al. (2021)	UK	Cross-sectional	Nonclinical 12 Autistic (ADOS positive) 36 Possibly autistic (traits)	153 No NDD	100% Male Age range 20–50 + IQ unknown (*screened with LDSQ*) Autistic Comorbidity: Depression (18.2%), GAD (18.2%), PTSD (9.1%), OCD (27.3%), Mania/hypomania (9.1%), Alcohol dependency (9.1%), Drug dependency (18.2%), SH (18.2%) Possibly Autistic Comorbidity: Depression (29.7%), GAD (27%), PTSD (16.2%), OCD (21.8%), Mania/hypomania (21.6%), Psychosis (8.1%), Alcohol dependency (21.6%), Drug dependency (32.4%), SH (18.9%)	ADI-R ADOS AQ-10 AQ-20	MINI (self-report) and ICD-10 SI: Past month A: Lifetime	Autistic SI: 3 (27.3%) SAB: 5 (45.5%) Possibly Autistic SI: 11 (30.6%) SAB: 24 (64.9%)
*Costa *et al*. (2020)*^*2*^	*Luxembourg*	*Cross-sectional*	*Nonclinical* *150 Autistic*	*189 Non-autistic*	*32.7% Male* *Age range 18–64 (M* = *33.74, SD* = *11.81)* *IQ unknown (without ID specified)* *Comorbidity: Depression (67%), Anxiety (38%), Other (39%)*	*AQ-S* *Self-identified formal diagnosis*	*SBQ-R (self-report)* *Lifetime*	*SBQ-R* ≥ *8: 94 (62.7%)*
*Dell'Osso *et al*. (2019)*^*2*^	*Italy*	*Cross-sectional*	*Nonclinical* *34 Autistic (ASC)* *68 Possibly autistic (traits)*	*160 Non-autistic*	*Autistic* *52.9% Male (n* = *16)* *Age (M* = *29.8, SD* = *12.1)* *Comorbidity: Anxiety (17.8%), Eating Disorder (5.9%)* *Possibly Autistic* *70.6% Male (n* = *20)* *Age (M* = *21.5, SD* = *3.1)* *Comorbidity: Anxiety (33.8%), Bipolar (8.8%) Eating Disorder (11.8%)* *IQ unknown (without ID specified)*	*AdAS Spectrum* *DSM-V*	*MOODS-SR lifetime version (self-report)* *Lifetime*	*Autistic* *Overall: 12 (35.3%)* *Possibly Autistic* *Overall: 29 (42.6%)*
Demirkaya et al. (2016)	Turkey	Retrospective chart review	Clinical 55 Autistic	-	89% Male Age range 7–20 (M = 13.56, SD = 2.9) IQ range 70–126 (M = 93.53, SD = 14.52; WISC-R, Cattell Scale, Stanford-Binet Scale) Comorbidity: Depression (18.2%), Anxiety (43.6%), OCD (18.2%), Bipolar Disorder (5.5%), Psychotic Features (9.1%), ADHD (65.5%), Self-mutilation (34.5%)	Medical records DSM-IV-TR	Eskin's Suicide Screening Questionnaire (self-report) SI: Past 12 months SAB: Lifetime	SI: 9 (16.3%) SAB: 7 (12.7%)
Dow et al. (2021)	US	Cross-sectional	Nonclinical 98 Autistic	-	68.4% Male Age (M = 28.2, SD = 10.9) IQ unknown (*lack of legal guardianship as proxy for intellectual functioning*) Comorbidity: Depression (55.1%), Panic, stress or worrying (63.3%) Employed: 36.7%	Documented proof	DSI-SS (self-report) SI/SP: Past 2 weeks SA: Lifetime	SI: 12 (12.2%) SP: 2 (2.1%) SAB: 19 (19.4%)
Green et al. (2000)	UK	Cross-sectional	Clinical 20 Autistic	20 Conduct Disorder	100% Male Age range 11–19 (Mdn = 13.75) IQ range 71–141 (M = 92.15, SD = 17.70; WISC, WAIS) Comorbidity: MDD (5%), Dysthymia (25%), GAD (35%), OCD (25%), Hyperkinetic (5%)	ADI ADOS ICD-10	IOWS (self-report) Past 3 months	SI: 2 (10%)
Greger et al. (2015)	Norway	Cross-sectional	Nonclinical 75 Possibly autistic	237 exposed to maltreatment/ 98 no maltreatment	46.7% Male Age range 12–20 (M = 16.9) IQ (M = 87.8; *met criteria for AS*) Comorbidity: Depression (52%), Anxiety (50.6%), ADHD (34.7%)	ASDI DSM-IV	CAPA (informant-report) SI/SP: Past 3 months SA: Lifetime	SI: 10 (13.3%) SP: 5 (6.9%) SAB: 27 (36%)
Hirvikoski et al. (2020)	Sweden	Case-cohort	Nonclinical 43,570 Autistic (Without ID)	4 groups (24,535 ASC without ID or ADHD/ 19,035 ASC with ADHD without ID/ 7704 ASC with ID without ADHD/ 2894 ASC with ID and ADHD) 270,840 age + sex matched controls without NDD	IQ unknown (*ID comparison group*) With ADHD 68.2% Male Age at SA (M = 21.99, SD = 8.91) Age at diagnosis (M = 19.45, SD = 11.70) Comorbidity: Depression (28.53%), Anxiety (27.83%), Bipolar disorder (6.61%), Schizophrenia (1.38%), EUPD (4.15%), SUD (15.49%) Without ADHD 67.9% Male Age at SA (M = 24.13, SD = 10.95) Age at diagnosis (M = 21.67, M = 15.38) Comorbidity: Depression (24.9%), Anxiety (22.69%), Bipolar Disorder (4.17%), Schizophrenia (4.87%), EUPD (2.45%), SUD (8.93%)	NPR ICD-10	NPR and ICD codes (observational) Lifetime	No ADHD SAB: 2066 (8.4%) ADHD SAB: 2397 (12.6%)
Hooijer & Sizoo (2020)	The Netherlands	Cross-sectional	Clinical 74 Autistic	-	60.8% Male Age range 23–42.3 (Mdn = 28.5) IQ unknown (*recruited through services for adults with average or higher intelligence*) Comorbidity: Depression (33.8%), Anxiety (16.2%), PTSD (4.1%), OCD (5.4%), Personality Disorder (6.8%), Substance use (6.8%)	ADI-R Education and mental health records DSM-IV	BSS (Dutch; self-report) SI: Past week SA: Lifetime	SI: 52 (70.3%) SAB: 21 (28.4%)
*Hu *et al*. (2019)*^*2*^	*Taiwan*	*Cross-sectional*	*Clinical* *219 Autistic*	*-*	*87.7% Male* *Age range 11–18 (M* = *13.7, SD* = *2.1)* *IQ range 80–127 (M* = *92.4, SD* = *10.9; WISC-IV)* *Comorbidity: Measured symptoms of Depression, Anxiety, ADHD and ODD*	*Formal diagnosis* *SRS (Chinese)* *DSM-V*	*KSADS-E—suicidality module (self-report)* *Past 12 months*	*45 (20.5%)*
Jackson et al. (2018)	US	Cross-sectional	Nonclinical 56 Autistic	-	46.4% Male Age range 18–57 (M = 22.98, SD = 6.01) Age range of diagnosis 3–38 IQ unknown (*post-secondary students*) Comorbidity: Depression (35.7%), GAD (33.9%), OCD (7.1%), Bipolar Disorder (5.4%), ADHD (23.2%)	AQ-10 Self-identified formal diagnosis	SBQ-R (self-report) Lifetime	SI: 11 (20%) SP: 22 (40%) SAB: 8 (14.5%)
Moseley et al. (2020)	UK	Cross-sectional	Nonclinical 102 Autistic (77 Self-harmers/ 25 Non-self-harmers)	-	28.4% Male Age range 54 (M = 42.26, SD = 13.91) Age at diagnosis (M = 34, SD = 17.2) IQ unknown (*educated to at least GCSE/ late diagnosis corroborates cognitive level*) Comorbidity: Depression (59.8%), Anxiety Disorders (GAD, social anxiety, OCD, specific phobias, PTSD; 53%), ADHD (13%), SH (75.5%) Employed: full-time (50%), voluntary (11.8%)	Self-identified formal diagnosis	SBQ-R (self-report) Lifetime *Item "Have you ever thought about or attempted to kill yourself?"*	SI: 30 (29.4%) SP: 36 (35.3%) SAB: 29 (28.4%)
Moses (2018)	US	Cross-sectional	Nonclinical 159 Autistic	10,330 TD/ 2873 youth with ≥ 1 disability	Information for whole “disabled” sample 49.4% Male Age 12–18 + (M = 15.9, SD = 1.2) IQ unknown (*self-report used, high school students*) Comorbidity: ≥ 1 type of disability (Emotional/ Mental health problems, Autism Spectrum, Hearing impairment, Vision impairment, Physical/ Mobility, Learning disability, Speech/ language problem, health problem, ADHD)	Self-identified	Unstandardised survey (self-report) Past 12 months *“During the past 12 months, have you attempted to kill yourself?” (No; Yes, 1 time; Yes, more than 1 time)*	SAB: 29 (18.2%)
Mukaddes & Fateh (2010)	Turkey	Cross-sectional	Clinical 37 Autistic	-	86.5% Male Age range 6–20 (M = 10.9, SD = 4.5) IQ range 90–139 (M = 116, SD = 14; WISC-R) Comorbidity: MDD (29%), GAD (5.4%), OCD (32%), Bipolar Disorder (8%), Eating Disorder (2.7%), Substance use (2.7%), ADHD (45%) Employed: voluntary (44%)	Developmental history Psychiatric interviews DSM-IV	Unstandardised clinician led interview (self-report) Unspecified	SAB: 6 (16%)
Paquette-Smith et al. (2014)	Canada	Cross-sectional	Nonclinical 56 Autistic	-	44% Male Age range 18–61 (M = 34.5, SD = 11.2) IQ unknown (*diagnosed AS*) Comorbidity: Depression (88%), Anxiety (94%),	AQ Self-identified formal diagnosis	Unstandardised (self-report) Unspecified *Asked if they had ever attempted suicide*	SAB: 18 (36%)
Pelton et al. (2020)	UK	Cross-sectional	Nonclinical 350 Autistic	339 Non-autistic	35.4% Male Age range 18–90 (M = 41.91, SD = 13.59) IQ unknown (*AQ requires average or above average intelligence*) Comorbidity: ≥ 1 mental health condition (68.6%) Employed: full-time (30.3%)	AQ-S Self-identified formal diagnosis	SBQ-R (self-report) Lifetime	SI: 55 (16.1%) SP: 140 (40.9%) SAB: 131 (38.3%)
Pilunthanakul et al. (2021)	Singapore	Cross-sectional	Clinical 101 Autistic	-	87.1% Male Age range 9—18 (M = 14.6, SD = 2.3) IQ unknown (*co-occurring ID excluded*) Comorbidity: MDD (19%)	Formal diagnosis DMS-V ICD-10	PHQ-9 (self-report AND informant-report) SI: Past month SA: Lifetime *Items "has had serious thoughts about ending life in the past month" and "has made a suicide attempt at least once in whole life"*	Self-report SI: 17 (16.8%) *Parent-report SI: 9 (8.9%)*^*2*^ Self-report SAB: 23 (22.8%) *Parent-report SAB: 14 (13.9%)*^*2*^
Raja et al. (2011)	Italy	Retrospective chart review	Clinical 19 Autistic	-	94.7% Male Age range 18—56 (M = 29.84, SD = 10.39) IQ (M = 88.74, SD = 14.59; ID removed and recalculated by VN) Comorbidity: Schizophrenia (63.2%), Alcohol abuse (15.8%), Mania with psychotic signs (5.3%), Depression (21.1%), OCD (10.5%)	Clinical assessments Medical records Psychiatric interviews DSM-IV-TR	Unstandardised (self-report) Past month *Asked whether they had: a) wished to die, b) thought about suicide in general, c) thought about methods for possible suicide, d) attempted suicide or self-harmed in any way*	SI: 37 (48%) SP: 9 (12%) *SAB: 1 (1%)*^*2*^
*Ryden *et al*. (2008)*^*2*^	*Sweden*	*Cross-sectional*	*Clinical* *6 Autistic*	*35 Non-autistic with BPD*	*0% Male* *Age (M* = *31.2, SD* = *8.89)* *IQ (M* = *98.7, SD* = *18.8; WAIS-III)* *Comorbidity: BPD (100%), MDD, Panic disorder, PTSD, GAD, Eating disorder, Bipolar Disorder, OCD, Psychotic episodes (% unknown)* *Employed: 16.7%*	*ASDI* *FTF A-TAC* *DSM-IV*	*SUAS (self-report)* *Lifetime*	+ *5 SA: 3 (50%)*
Sharpley et al. (2016)	Australia	Cross-sectional	Nonclinical 39 Autistic	-	0% Male Age range 6–17 (M = 10.1, SD = 2.7) IQ threshold > 70 (WASI-II)	ADOS-2 DSM-IV-TR DSM-V	CASI-MDD (self-report) Unspecified *Item 28 "I talk about dying and killing myself"*	SI: 14 (35.8%)
Shtayermman (2020)	US	Cross-sectional	Nonclinical 14 Autistic	-	100% Male Age range 15–24 (M = 16.6, SD = 1.5) IQ unknown (*diagnosed AS*) Comorbidity: MDD (28.6%), GAD (15.4%)	Self-identified formal diagnoses DSM-IV-TR	SIQ Adolescent version (self-report) Past month	SI: 3 (21.4%)
Shtayermman (2008)	US	Cross-sectional	Nonclinical 10 Autistic	-	90% Male Age (M = 19.7, SD = 3.0) IQ unknown (*diagnosed AS*) Comorbidity: MDD (20%), GAD (30%) Employed: 40%	KADI	SIQ (self-report) Past month	SI: 5 (50%)
South et al. (2020)	US	Cross-sectional	Nonclinical 26 Autistic (previous diagnosis) 48 Possibly autistic (traits)	-	0% Male Age range 18–49 (M = 25.42, SD = 7.25) IQ range 83–140 (M = 114.8, SD = 11.12; FSIQ-2) Comorbidity: Mild/great severity of depression (76%), Mild/great severity of anxiety (81%) Employed: part-time (66.2%), full-time (6.8%)	ADOS AQ BAPQ SRS-2	SBQ-R (self-report) Lifetime SI: Past 12 months	Autistic *SBQ-R* ≥ *8: 13 (50%)* SI: 10 (39%) SP: 13 (50%) SAB: 4 (15.4%) Possibly Autistic *SBQ-R* ≥ *8: 17 (35.4%)* SI: 19 (40%) SP: 9 (19%) SAB: 8 (16.7%)
Storch et al. (2013)	US	Cross-sectional	Clinical 102 Autistic	-	77% Male Age range 7–16 (M = 10.55, SD = 2.31) IQ threshold ≥ 70 Comorbidity: Depression (13.7%), GAD (74.5%), PTSD (5.9%), OCD (35.3%)	ADI-R ADOS DSM-IV-TR	ADIS-IV-C/P–Major Depressive Modules (self-report AND informant-report) SI/SP: Current SA: Lifetime	Child-report SI: 13 (13%) *Parent-report SI: 12 (12%)* Child-report SP: 9 (9%) *Parent-report SP: 5 (5%)* Child-report SAB: 1 (1%) *Parent-report SAB: 1 (1%)*
Strang et al. (2021)	US	Cross-sectional	Both & TGNC 54 Autistic (27 Cisgender; 27 Transgender) 13 Possibly utistic (Transgender subthreshold autism)	26 SAAB and age matched transgender allistic	Cisgender Autistic 59% Male Age range 13—21 (M = 16.42, SD = 1.9) IQ (M = 53.91, SD = 9.33; Matrix reasoning) Transgender Autistic 59% Male; 41% AMAB Age range 13—21 (M = 17.37, SD = 2.08) IQ (M = 112.73, SD = 15.89; WASI-2, WISC-5, WAIS-5) Transgender Possibly Autistic 62% Male; 38% AMAB Age range 13—21 (M = 16.99, SD = 2.14) IQ (M = 117.54, SD = 17.4; WASI-2, WISC-5, WAIS-5)	ADI-R ADOS-2 DSM-IV	ASEBA-YSR (self-report) Past 6 months *Item 91 "I think about killing myself"*	Autistic Cisgender SI: 7 (25.9%) Transgender SI: 15 (55.5%) Possibly Autistic Transgender SI: 9 (69.2%)
Strauss et al. (2021)	Australia	Cross-sectional	Nonclinical & TGNC 172 Autistic	687 Non-autistic Transgender	38.8% Male Age range 14–25 IQ unknown (*self-report used*) Comorbidity: Depression (91.3%), Anxiety (93.6%), PTSD (57%), Eating Disorder (52.3%), Psychosis (51.2%), Personality Disorder (55.2%), SUD (41.3%), SH (85.1%)	Self-identified formal diagnosis	Unstandardised (self-report) Lifetime *Information on reckless behaviour that purposely puts one’s life at risk, suicidal thoughts, and suicide attempts*	SI: 135 (87.7%) SAB: 87 (57.2%)
Takara & Kondo (2014a)	Japan	Case–control	Clinical 70 Autistic	360 Non-autistic	50% Male Age range 18–55 (M = 30.2, SD = 10.1) IQ unknown (*without apparent intellectual problems; high school education or above; JART*) Comorbidity: MDD (30%), Bipolar Disorder (22.9%%), Psychotic-like experiences (2.9%)	AQ (Japanese) Confirmation from parents Medical records DSM-IV-TR	Unstandardised (self-report) Unspecified *Presence/absence of "suicide-related behaviours" confirmed by patients*	SAB: 17 (24%)
Takara & Kondo (2014b)	Japan	Case–control	Clinical 37 Autistic	299 Non-autistic	45.95% Male Age range 18–55 (M = 28.5, SD = 8.8) IQ unknown (*without apparent intellectual problems; receiving mainstream education*) Comorbidity: Depression (100%), Bipolarity (43.2%), Psychotic features (5.4%), Cluster B Personality Disorder (8.1%), Substance use (8.1%) Employed, homemaker or graduate: 75.7%	AQ (Japanese) Confirmation from parents Medical records DSM-IV	Unstandardised (observational) Unspecified *Patients visiting outpatient clinic after suicide attempts*	SAB: 9 (24.3%)
Umeda et al. (2021)	Japan	Cross-sectional	Nonclinical 113 Possibly autistic (traits)	2227 assessed for ASC/ 2297 assessed for ADHD	7.1% Male Age range 20–75 IQ unknown (*AQ requires average or above average intelligence*) Comorbidity: MDD (6.2%), Dysthymia (0.9%), Anxiety (7.1%), Bipolar (0.5%), Substance use (15.6%) Employed: 4.9%	AQ-10 (Japanese) DSM-IV	Unstandardised (self-report) Lifetime *Asked whether they had ever seriously thought about committing suicide*	SI: 17 (15%)
Wijnhoven et al. (2019)	The Netherlands	Cross-sectional	Clinical 93 Autistic	-	76.3% Male Age 8–15 (M = 11.15, SD = 1.98) IQ (M = 102.16, SD = 18.14)	ADOS DSM-V	Child Depression Inventory (CDI-2) Dutch version Current *Item 9 “I do not think about killing myself/ I think about killing myself but would not do it/ I want to kill myself”*	SI: 31 (34.4%)
Zhou et al. (2018)	China	Cross-sectional	Nonclinical 39 Possibly autistic (traits) 2780 students	-	41.03% Male Age range 18–30 IQ unknown (*college students*) Comorbidity: Depression (53.8%), Mild anxiety (5.1%), OCD (71.8%)	AQ (Chinese)	Unstandardised (self-report) Unspecified *Asked about suicide ideation, suicide plans, and suicide attempts (e.g. Do you have, or did you ever have a suicide plan?)*	SI: 12 (30.8%) SP: 4 (10.3%)

Not included in qualitative synthesis due to: ^1^presence of ID in group, ^2^did not report separate suicidality outcomes/ outcome unable to be quantitatively synthesised

*ASEBA-YSR* Achenbach System of Empirically Based Assessment-Youth Self-Report, *AAA* Adult Asperger Assessment, *AdAS Spectrum* Adult Autism Subthreshold Spectrum, *ASRI* Adult Self-Report Inventory, *ADIS-IV-C/P* Anxiety Disorder Interview Schedule—Child and Parent Versions, *ASDI* Asperger Syndrome Diagnostic Interview, *AS* Asperger’s Syndrome, *AMAB* Assigned Male at Birth, *ADHD* Attention Deficit Hyperactivity Disorder, *ADI* Autism Diagnostic Interview, *ADOS* Autism Diagnostic Observation Schedule, *AQ-S* Autism Quotient-Short version, *AQ* Autism Quotient, *ASC* Autism Spectrum Condition, *ASSQ* Autism Spectrum Screening Question, *BSS* Beck Scale for Suicidal Ideation, *BASC-2 PRS-C* Behaviour Assessment for Children Second Edition, *BPD* Borderline Personality Disorder, *BAPQ* Broad Autism Phenotype Questionnaire, *CAPA* Child and Adolescent Psychiatric Assessment, *DSI-SS* Depressive Symptom Inventory—Suicidality Subscale, *DSM* Diagnostic and Statistical Manual of Mental Disorders, *EUPD* Emotionally Unstable Personality Disorder, *FTF A-TAC* “Five-to-Fifteen”-Autismtics, ADHD and other comorbidities interview, *FSIQ* Full Scale Intelligence Quotient, *GD* Gender Dysphoria, *GAD* Generalised Anxiety Disorder, *IQ* Intelligence Quotient, *ICD* International Classification of Diseases, *IOWS* Isle of Wight Subject Interview, *JART* Japanese version of the Adult Reading Test, *KSADS-E* Kiddie Schedule for Affective Disorders and Schizophrenia-Epidemiological version, *KADI* Krug Asperger's Disorder Index, *LDSQ* Learning Disability Screening Questionnaire, *MDD* Major Depressive Disorder, *MINI* Mini International Neuropsychiatric Interview, *NDD* Neurodevelopmental Difficulties, *NPR* National Patient Register, *NSSI* Non-suicidal Self-Injury, *OCD* Obsessive Compulsive Disorder, *ODD* Oppositional Defiant Disorder, *PHQ* Patient Health Questionnaire, *PTSD* Post-traumatic stress-disorder, *SH* Self-harm, *SRS* Social Responsiveness Scale, *SUD* Substance Use Disorder, *SI* Suicidal Ideation, *SIQ* Suicidal ideation Questionnaire, *SUAS* Suicide Assessment Scale, *SAB* Suicide Attempts and Behaviours, *SBQ-ASC* Suicide Behaviours Questionnaire-Autism Spectrum Conditions, *SBQ-R* Suicide Behaviours Questionnaire-Revised, *SP* Suicide Plans, *CASI-MDD* The Child and Adolescent Symptom Inventory-Major Depressive Disorder subscale, *MOODS-SR* The Mood Spectrum, self-report, *WASI* Wechsler Abbreviated Scale of Intelligence, *WAIS* Wechsler Adult Intelligence Scale, *WISC* Wechsler Intelligence Scale for Children

Primary studies covered 16 countries worldwide, with 50% in Europe (France *k* = 1; Italy *k* = 2; Luxembourg *k* = 1; Norway *k* = 1; The Netherlands *k* = 3; Sweden *k* = 2; Turkey *k* = 2; UK *k* = 8), followed by 22.5% in North America (US *k* = 8; Canada *k* = 1); 20% in Asia (China *k* = 1; Japan *k* = 3; Korea *k* = 1; Singapore *k* = 1; Taiwan *k* = 2) and 7.5% in Oceania (Australia *k* = 3). The main design utilised was cross-sectional (*k* = 33; 82.5%), along with a small number of retrospective chart reviews (*k* = 2; 5%), case–control studies (*k* = 3; 7.5%), one cohort (2.5%), and one intervention (2.5%). Most reported prevalence estimates for suicidal ideation (*k* = 29; 72.5%), followed by suicide attempts and behaviours (*k* = 26; 65%), with fewer reporting on suicide plans (*k* = 8; 20%). Five studies gave an estimate of overall suicidality or the proportion of those at risk of suicidality using a standardised measure (12.5%).

To ascertain autism or possible autism, 60% of primary studies utilised a validated screening tool for non-diagnostic purposes (e.g. AQ), or extracted autism diagnoses from medical records (*k* = 24); 27.5% employed validated diagnostic assessments (e.g. Autism Diagnostic Observation Schedule) to confirm autism diagnosis (*k* = 11); and 12.5% determined autism or possible autism based solely on self- or informant-report (*k* = 5). Conversely, 40% of primary studies used a measurement tool specifically validated for suicidality or record linkage (*k* = 16); 27.5% utilised a general validated measurement tool which included items or modules relevant to suicidality or extracted relevant information from medical records (*k* = 11); and the remainder used unstandardised questions or did not specify exactly how suicidality was measured (*k* = 13; 32.5%).

Three primary studies (7.5%; 1 intervention study and 2 retrospective chart reviews) were not assessed for quality as there were no corresponding versions of the NOS for these study designs. Of the 37 that were assessed, five primary studies were classified at high risk of bias based on the adapted NOS score (13.5%), 28 at an unclear risk (75.7%), and four at a low risk (10.8%). Studies mainly failed to justify sample size (*k* = 28; 75.7%) or did not report adequate response or participation rates (*k* = 27, 73%).

From the 40 included primary studies, 36 of these were able to be numerically synthesised. Four primary studies were not included in this synthesis because they did not provide separate estimates of suicidality outcomes [12, 71, 72] or only provided an estimate for multiple suicide attempts [73]. The population of these four studies represented 506 autistic and 68 possibly autistic individuals without co-occurring ID, which made up 1.2% of the overall population in the review*.*

### Meta-analysis

**Fig. 2:**
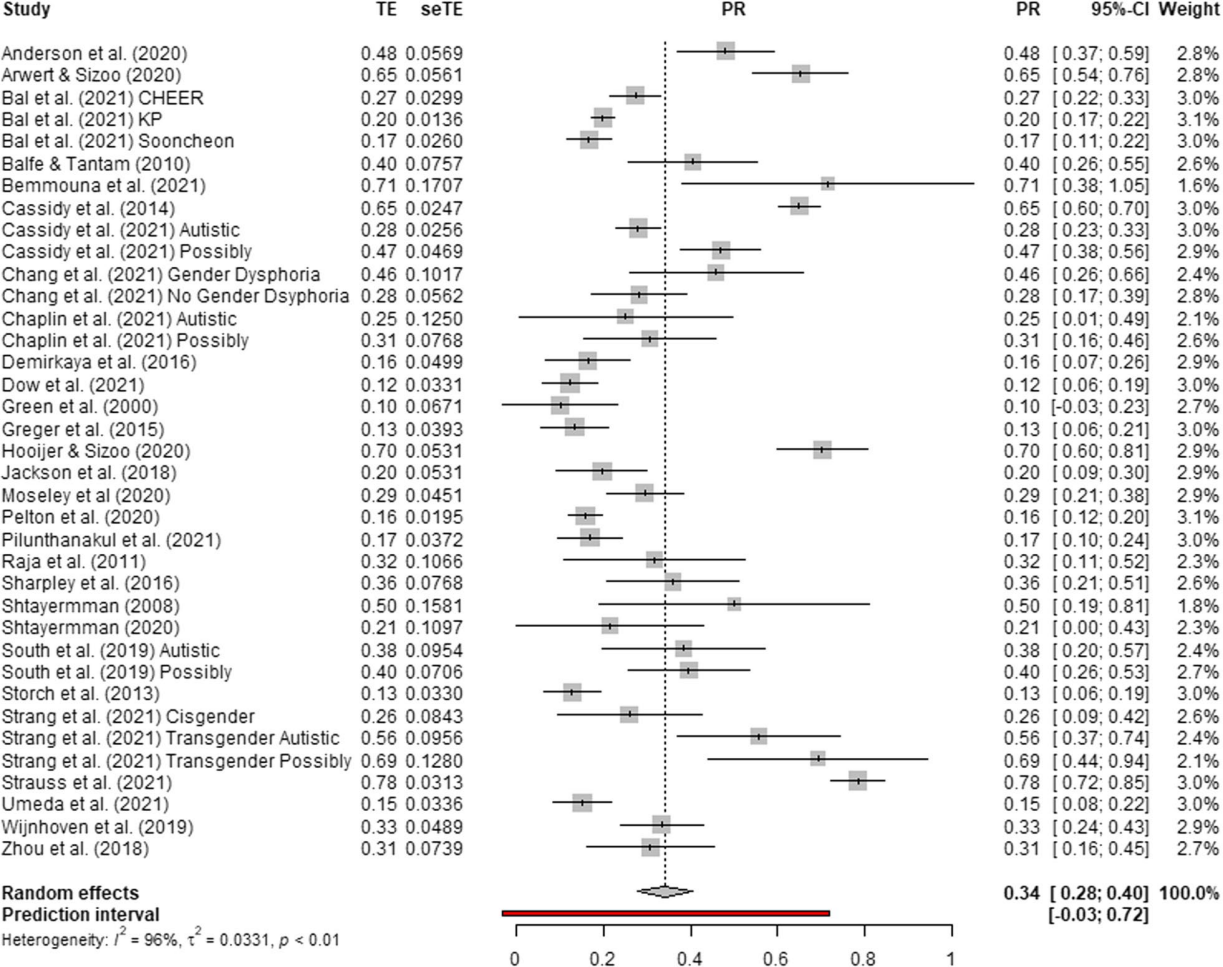
Forest plot of suicidal ideation prevalence in autistic and possibly autistic people

**Fig. 3:**
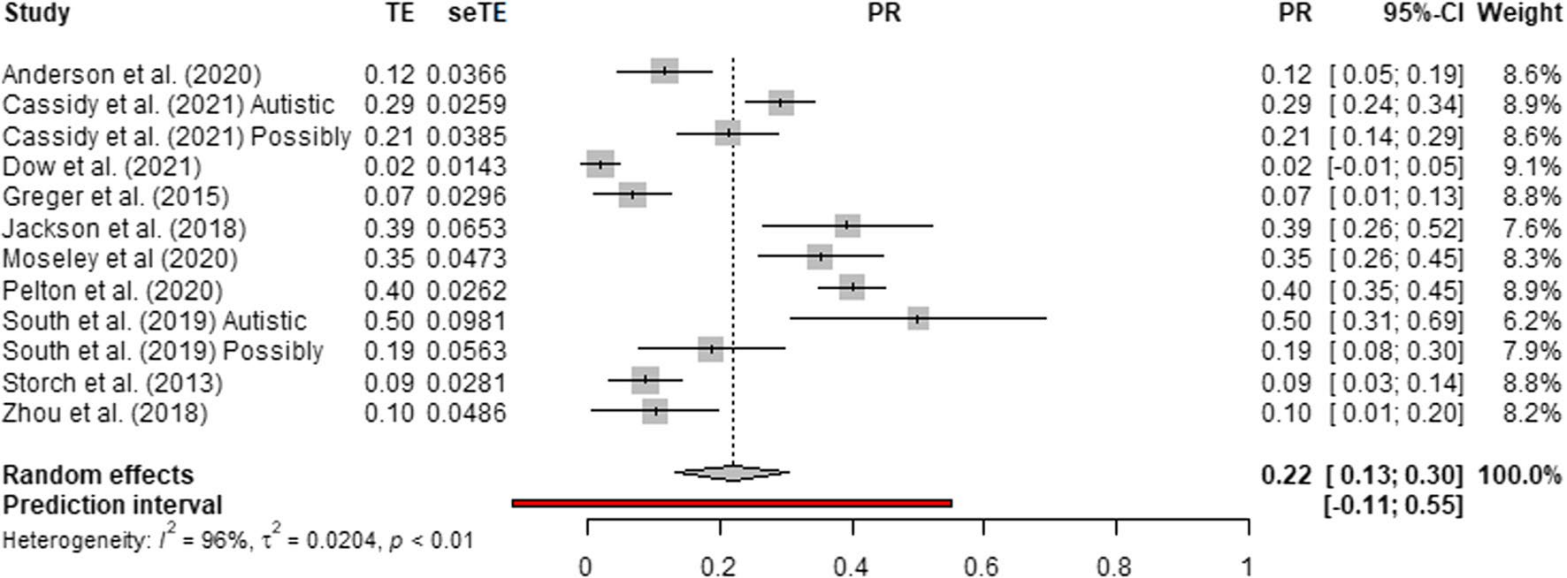
Forest plot of suicide plan prevalence in autistic and possibly autistic people

**Fig. 4:**
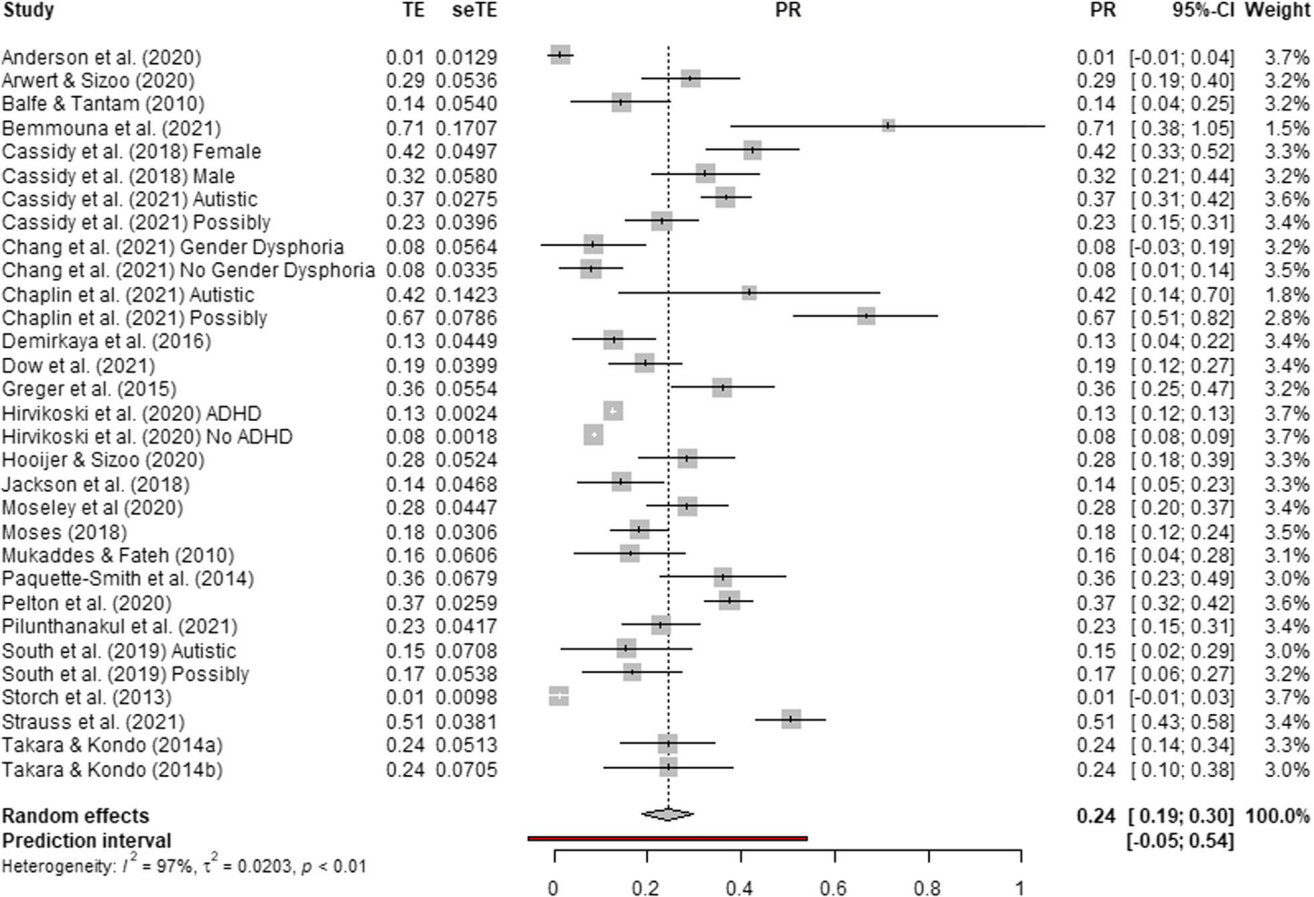
Forest plot of suicide attempts and behaviours prevalence in autistic and possibly autistic people

All three random effects models yielded significant pooled prevalence estimates of suicidality among autistic and possibly autistic individuals without co-occurring ID. Pooled prevalence estimates were 34.2% for suicidal ideation (95% CI 27.9%; 40.5%, *p* < 0.001, *I*^2^ = 96.5%, *τ*^2^ = 0.033) with a 95% prediction interval (-3.3%; 72.7%), seen in Fig. 2; 21.9% for suicide plans (95% CI 13.4%; 30.4%, *p* < 0.001, *I*^2^ = 95.9%, *τ*^2^ = 0.020) with a 95% prediction interval (11.4%; 55.2%), seen in Fig. 3; and 24.3% for suicide attempts and behaviours (95% CI 18.9%; 29.6%, *p* < 0.001, *I*^2^ = 96.7%, *τ*^2^ = 0.020) with a 95% prediction interval (-5.4%; 53.9%), seen in Fig. 4. High levels of heterogeneity (*I*^2^ > 75) [61] were observed in all three analyses, indicating estimates of prevalence may be biased by the presence of uncontrolled or confounding factors. TE, seTE, prevalence rate, confidence intervals, prediction intervals and weighting by the random effects model are reported in Figs. 2, 3 and 4.

Given substantial heterogeneity, sensitivity analyses of random effects models were conducted. Baujat plots revealed estimates with a potential disproportional influence were present in the random effects model for suicidal ideation [74], suicide plans [75–77], and suicide attempts and behaviours [78]. Omission of these using “leave-one-out” analyses did not reveal any significant changes in the overall meta-analytic effect, with 95% CIs still substantially overlapping with main results across all analyses (see Additional file 1: Supplementary Materials 5).

Visual inspection of the funnel plot and Egger’s regressions indicated possibility of publication bias and small study effects in the distribution of prevalence estimates for suicide attempts and behaviours; however, trim and fill procedures yielded no corrections (see Additional file 1: Supplementary Materials 6). Orwin’s method [68] indicated 220 studies with a null effect would be required to reduce the observed pooled prevalence of suicide attempts and behaviours to that of the general population [31, 79] suggesting the observed effect is robust to publication bias.

### Moderator analysis

Subsequent analyses focused on identifying sources of heterogeneity between prevalence estimates of suicidal ideation, suicide plans and suicide attempts and behaviours in the primary studies. Subgroup analyses were carried out for categorical covariates (see Table 4). This revealed geographical location (*p* = 0.005), transgender or gender non-conforming samples (*p* < 0.001) and type of report (*p* < 0.001) significantly moderated suicidal ideation. Prevalence estimates were higher in samples of transgender or gender non-conforming participants (63.8%) compared to samples that were not (30.8%), and higher when measures used self-report (36.7%) compared to informant-report (19.5%). Post hoc comparisons of geographical location also indicated prevalence estimates of suicidal ideation were significantly lower in Asia (21.3%) vs Europe (37.8%; *p* = 0.012), and Asia vs Oceania (54.86%, *p* = 0.012).

**Table 4:** Subgroup analyses for categorical moderators of prevalence estimates

Subgroups	*k*	Prevalence (95% CI)	Heterogeneity Analysis						Between-subgroups Differences		
			*p*	*QE*	*df*	*p*	*τ*^2^	*I*^2^	*QM*	*df*	*p*
*Suicidal Ideation*											
Group											
Autistic	27	.3607 (.2824; .4390)	< .001	693.67	26	< .001	.038	96.3%	1.45	1	.228
Possibly Autistic	10	.2872 (.1969; .3775)	< .001	69.74	9	< .001	.018	87.1%			
Age Group											
Youth (< 20 years)	18	.3082 (.2159; .4006)	< .001	386.94	17	< .001	.035	95.6%	1.05	1	.307
Adult (≥ 20 years)	19	.3738 (.2887; .4588)	< .001	431.37	18	< .001	.031	95.8%			
Geographical Location											
Asia	8	.2207 (.1732; .2681)	< .001	21.32	7	.003	.003	67.2%	12.79	3	.005 **
Europe	16	.3639 (.2627; .4651)	< .001	396.97	15	< .001	.038	96.2%			
North America	10	.3209 (.2050; .4369)	< .001	54.44	9	< .001	.027	83.5%			
Oceania	3	.5485 (.2972; .7998)	< .001	40.89	2	< .001	.046	95.1%			
Setting											
Clinical	11	.3523 (.2171; .4875)					.047	93.8%	1.62	2	.444
Nonclinical	23	.3220 (.2484; .3956)	< .001	673.11	22	< .001	.029	96.7%			
Both	3	.4902 (.2382; .7422]					.039	79.8%			
TGNC Sample											
Yes	4	.6378 (.4808; .7949)	< .001	13.56	3	.004	.018	77.9%	14.81	1	< .001 ***
No	33	.3087 (.2500; .3673)	< .001	561.28	32	< .001	.025	94.3%			
Suicidality Measure											
Suicidality	13	.3429 (.2368; .4491)	< .001	197.64	12	< .001	.034	97.0%	5.95	2	.051
General	16	.2670 (.2011; .3330)	< .001	69.12	15	< .001	.014	78.3%			
Unstandardised	8	.4709 (.3123; .6295)	< .001	234.63	7	< .001	.046	93.9%			
Type of Report											
Self	33	.3626 (.2945; .4306)	< .001	740.70	32	< .001	.034	71.8%	14.38	1	< .001 ***
Informant	4	.1950 (.1414; .2486)	< .001	10.65	3	.014	.002	95.7%			
Risk of bias											
Low risk	6	.3651 (.2874; .4427)	< .001	16.26	5	.006	.006	69.2%	0.28	1	.594
Any risk (unclear or high)	28	.3352 (.2576; .4129)	< .001	817.93	27	< .001	.040	96.7%			
*Suicide Plansª*											
Group											
Autistic	8	.2606 (.1421; .3791)	< .001	254.18	7	< .001	.027	97.2%	2.97	1	.085
Possibly Autistic	4	.1386 (.0665; .2108)	< .001	10.25	3	.017	.004	70.7%			
Age Range											
Youth (< 20 years)	2	.0789 (.0390; .1188)	< .001	0.23	1	.628	0	0.0%	10.87	1	.001 **
Adult (≥ 20 years)	10	.2492 (.1561; .3422)	< .001	251.74	9	< .001	.020	96.4%			
Geographical Location^b^											
Europe	5	.2646 (.1490; .3802)	< .001	77.14	4	< .001	.016	94.8%	0.15	1	.701
North America	5	.2238 (.0511; .3966)	.011	59.96	4	< .001	.036	93.3%			
Suicidality Measure											
Suicidality	8	.2850 (.1807; .3893)	< .001	248.31	7	< .001	.020	97.2%	13.12	2	.001 **
General	2	.0789 (.0390; .1188)	< .001	0.23	1	.628	0	0.0%			
Unstandardised	2	.1117 (.0544; .1690)	< .001	0.06	1	.814	0	0.0%			
Risk of bias											
Low risk	4	.2762 (.1727; .3796)	< .001	10.60	3	< .001	.008	71.7%	1.28	1	.258
Any risk (unclear or high)	8	.1890 (.0788; .2992)	< .001	209.08	7	< .001	.024	96.7%			
*Suicide Attempts and Behaviours*											
Group											
Autistic	26	.2227 (.1689; .2765)	< .001	816.84	26	< .001	.017	96.8%	1.31	1	.253
Possibly Autistic	4	.3497 (.1386; .5608)	.001	32.12	3	< .001	.043	90.7%			
Age Group											
Youth (< 20 years)	9	.1916 (.0891; .2941)	< .001	227.86	8	< .001	.028	96.5%	1.41	1	.235
Adult (≥ 20 years)	22	.2643 (.2019; .3267)	< .001	663.16	21	< .001	.019	96.8%			
Geographical Location											
Asia	5	.1700 (.0923; .2478)	< .001	14.40	4	.006	.005	72.2%	6.91	3	.075
Europe	16	.2907 (.2156; .3658)	< .001	604.82	16	< .001	.021	97.4%			
North America	7	.1632 (.0862; .2401)	< .001	80.27	6	< .001	.009	92.5%			
Oceania	2	.2581 (− .2249; .7410)	.295	149.95	1	< .001	.121	99.3%			
Setting											
Clinical	11	.1880 (.1154; .2606)	< .001	119.06	10	< .001	.012	91.6%	2.13	1	.144
Nonclinical	20	.2680 (.1987; .3374)	< .001	755.19	19	< .001	.023	97.5%			
TGNC Sample											
Yes	2	.2966 (− .1174; .7106)	.160	38.5	1	< .001	.087	97.4%	0.09	1	.768
No	28	.2372 (.1846; .2898)	< .001	789.80	28	< .001	.018	96.5%			
Suicidality Measure											
Suicidality	16	.2439 (.1897; .2980)	< .001	541.89	15	< .001	.010	96.1%	0.07	2	.963
General	6	.2340 (.0344; .4336)	.022	102.18	5	< .001	.058	95.1%			
Unstandardised	9	.2601 (.1418; .3784)	< .001	207.08	8	< .001	.029	97.2%			
Type of Report											
Self	27	.2506 (.1911; .3101)	< .001	668.54	26	< .001	.022	96.1%	0.71	1	.399
Other	4	.1923 (.0706; .3140)	.002	221.36	3	< .001	.014	98.6%			
Risk of bias											
Low risk	6	.1876 (.1011; .2740)	< .001	302.95	5	< .001	.010	98.3%	1.45	1	.228
Any risk (unclear or high)	23	.2538 (.1894; .3182)	< .001	585.11	22	< .001	.022	96.2%			

*TGNC* Transgender or gender non-conforming

ªInsufficient estimates for ‘TGNC’ and ‘Type of Report’ subgroups

^b^Insufficient estimates for Asia (k = 1) Oceania (k = 1) and too different to combine

*k* = No of estimates; CI = Confidence Interval; QE = Test of Residual Heterogeneity; QM = Test of moderators

*p* = significant at * < .05, ** < .01, *** < .001

Age group (*p* = 0.001) and suicidality measures (*p* = 0.001) significantly moderated suicide plans. Prevalence estimates were higher in autistic adults (22.9%) compared to autistic youth (7.9%). Post hoc comparisons of suicidality measures indicated prevalence estimates of suicide plans were higher when using a tool specific to suicidality (28.5%) vs a general measure (7.9%; *p* < 0.001) or vs an unstandardised measure (11.2%; *p* = 0.004). No significant moderators were demonstrated for prevalence estimates of suicide attempts and behaviours using subgroup analyses.

Univariate meta-regressions were also carried out for continuous covariates (see Table 5). Proportion of male participants was a significant moderator for suicide plan prevalence only, accounting for over a third of the proportion of variance in the prevalence estimate (*R*^2^ = 35.5%), with a decrease of 0.4036 in the proportion of male participants for every unit change of suicide plan prevalence. Neither year of publication nor NOS total score as covariates significantly impacted on the results across any of the analyses.

**Table 5:** Univariate meta-regression analyses for continuous moderators of prevalence estimates

Covariates	*k*	Coefficient (95% CI)	SE	*z*	Heterogeneity Analysis						Test of Moderators		
					*QE*	*df*	*p*	τ^2^	I^2^	*QM*	*df*	*p*	R^2^
*Suicidal Ideation*													
Male (%)	36	− .145 (− .370; .081)	.115	− 1.258	823.245	34	< .001	.032	95.09%	1.5814	1	.207	2.07%
Year of Publication	37	.007 (− .007; .021)	.007	0.950	840.160	35	< .001	.033	95.27%	0.9023	1	.342	0.00%
NOS Overall Score	34	− .010 (− .056; .035)	.023	− 0.451	709.112	32	< .001	.034	95.59%	0.2037	1	.652	0.00%
*Suicide Plans*													
Male (%)	12	− .404 (− .722; − .085)	.162	− 2.486	106.626	10	< .001	.013	92.00%	6.1805	1	.013 *	35.54%
Year of Publication	12	.021 (− .013; .054)	.017	1.206	262.725	10	< .001	.020	94.64%	1.4549	1	.228	3.64%
NOS Overall Score	12	.035 (− .014; .084)	.025	1.397	238.824	10	< .001	.019	94.90%	1.9514	1	.162	7.09%
*Suicide Attempts and Behaviours*													
Male (%)	29	− .106 (− .301; .090)	.100	− 1.061	693.523	27	< .001	.021	99.51%	1.1266	1	.289	2.53%
Year of Publication	31	.010 (− .006; .027)	.008	1.127	856.604	29	< .001	.020	99.44%	1.4798	1	.224	0.53%
NOS Overall Score	29	− .008 (− .039; .023)	.016	− 0.519	888.396	27	< .001	.020	99.46%	0.2693	1	.604	0.00%

*k* No of estimates, *CI* Confidence Interval, *SE* Standard Error, *QE* Test of Residual Heterogeneity, *QM* Test of moderators

*p* = significant at * < .05, ** < .01, *** < .001

## Discussion

The main aim of the current systematic review and meta-analysis was to synthesise prevalence estimates of suicidality in autistic people and possibly autistic people without co-occurring ID. From 40 primary studies, 36 of these were meta-analysed representing 48,692 autistic and possibly autistic participants. Moderator analyses were conducted to evaluate how study and participant level characteristics influenced the prevalence of suicidality outcomes. This is the first meta-analysis to synthesise data in autistic people and possibly autistic people without co-occurring ID across all ages and provides novel pooled prevalence estimates for outcomes of suicidal ideation, suicide plans, and suicide attempts and behaviours in both groups. Such findings have important clinical and scientific implications to understanding and preventing suicide. Moreover, the use of robust, stringent and standardised procedures in line with PRISMA guidelines [50] ensures the accuracy of estimates and enhances the validity of findings.

High pooled prevalence estimates were demonstrated across all three suicidality outcomes; suicidal ideation was prevalent in over a third (34.2%) of autistic and possibly autistic people without co-occurring ID; suicide plans were prevalent in 21.9%, and suicide attempts and behaviours in 24.3%. These estimates remain considerably higher than those in the general population. For example, cross-national prevalence of suicidal ideation in the general population is approximately 9%, and between 2 and 3% for suicide plans and suicide attempts and behaviours [31, 78]. The large difference between these rates compounds the evidence that autistic people are at a particularly increased risk of suicidality [10, 18–22]. Additionally, prevalence estimates of suicidal ideation, suicide plans and suicide attempts and behaviours were found to be comparable between autistic and possibly autistic groups. This finding adds weight to previous research showing that possibly autistic people are equally at risk of suicidality [47, 48], and therefore should also be included in research and clinical considerations going forward.

High levels of heterogeneity were observed in each of the random effects models (*I*^2^ = 95.9–96.7%) and so subgroup analyses and univariate meta-regressions were conducted. These analyses showed prevalence of suicidal ideation and suicide plans varied for certain participant and study level characteristics; however, this was not the case for suicide attempts and behaviours.

Firstly, prevalence of suicidal ideation was moderated by geographical location, transgender or gender non-conforming samples and type of report. Suicidal ideation was found to differ across geographical locations, with lower prevalence estimates in Asia compared to Europe and Oceania. This finding is interesting, considering around two-thirds of global deaths by suicide occur in Asia [80]. In the current review, the geographical location of Asia predominately consisted of East Asian countries (Korea, Taiwan, China, Singapore and Japan), where lower prevalence may be explained by a range of factors such as the criminalisation of suicide [81], stigma towards both mental health problems [82–84] and autism [85–87], and the importance of maintaining family reputation within collectivist Asian societies [88]. As such, it is possible that self-reported suicidality or an autism diagnosis/ autistic traits may not be an accurate reflection of reality. More research is needed to better understand the complexities of suicidality in autistic and possibly autistic people across Asia.

The current findings also suggest suicidal ideation is higher in autistic and possibly autistic samples who are transgender or gender non-conforming. This is unsurprising, as transgender and gender non-conforming individuals in the general population exhibit much higher rates of suicidal ideation and suicidal behaviour than their cisgender peers [89–91]. Along with this, autistic people are more likely to be gender diverse than non-autistic people [74, 92], and gender-diverse people are also more likely to be autistic [93]. It is therefore possible that the intersection of these two identities compounds the risk of suicidality, resulting in a higher prevalence estimate. There is a clear need for future studies to report on diverse gender identities to investigate this relationship further. Moreover, clinicians working with transgender or gender non-conforming people and/or autistic people should be made aware of this possible overlap and the associated risk, to appropriately screen for and manage suicidality [74].

In addition, prevalence of suicidal ideation was higher for self-report measures of suicidality compared to informant-report. The two primary studies that only utilised informant-report were those that included samples of autistic adolescents’ or children [94, 95]. Studies which have used both informant and self-report found there to be poor agreement between parents and their autistic youth, where parents seem to underreport on various psychiatric symptoms, including suicidality [29, 96, 97]. This suggests self-report may provide a more accurate reflection of autistic youth’s internal experiences of suicidality and highlights the need for corroborating accounts alongside informant-report when this method is utilised.

Secondly, prevalence of suicide plans was moderated by age group, measurement of suicidality, and proportion of male participants. Prevalence of suicide plans were higher in autistic or possibly autistic adults (age ≥ 20 years) than youth (age < 20 years), but these age moderation effects were not observed for suicidal ideation or suicide attempts and behaviours. Similarly, large population-based studies show incidence of suicide attempts in autistic people with and without co-occurring ID does not significantly differ with age [14]. This comparable prevalence of suicidal ideation and suicide attempts and behaviours across age groups may be accounted for by risk factors of suicidality that are experienced by autistic people throughout their lives (e.g. mental health problems) [6–9, 41]. Consequently, older individuals could be more likely to have a suicide plan but are no more likely to think about suicide or make an attempt than those who are younger. Despite this, there is currently no research exploring this developmental trajectory of suicidality in autism [28], and more is needed to accurately determine any relationships between age and suicidality.

Moreover, suicide plans were found to be the highest when using a measurement tool specific to suicidality compared to a general or unstandardised tool. We know that autistic people interpret and respond differently to items and measures validated for use in the general population [24]. However, most studies reporting suicide plans used the SBQ-R [98], with one using the adapted version of this: the SBQ-ASC [24]. It is possible that these measures which assess suicide plans are homogenous enough to be sensitive to prevalence differences, compared to the wider variation of assessment methods used to measure suicidal ideation and suicide attempts and behaviours.

However, the current review only somewhat supported previous evidence that suicidality is more prevalent in autistic females [13, 15]. Meta-regression results highlighted an association between the proportion of male participants and prevalence of suicide plans only, in that as the proportion of male participants decreased, the prevalence of suicide plans increased. Interestingly, all but two of the studies reporting suicide plans included predominately female participants [75, 96] and several also reported on other gender identities [24, 76, 99, 100], providing a more representative sample. Failure to detect this association in suicidal ideation and suicide attempts and behaviours may indicate other samples were not diverse enough in terms of gender to reliably explore it as a moderator.

Lastly, no significant moderators were found for suicide attempts and behaviours, suggesting comparable prevalence across the subgroups examined. Alternatively, it is possible that heterogeneity may be explained by other variables, such as age of diagnosis, unemployment, or the presence of non-suicidal self-injury [41]. These are suggested to be risk factors for suicidality in autistic people, but further investigation was not possible due to insufficient data in primary studies. Further research is warranted to determine which of these factors, if any, moderate prevalence estimates of suicide attempts and behaviours in those who are autistic or possibly autistic.

### Limitations

While the current review was robust and inclusive, it did have some limitations which should be acknowledged. One of these being that 91.7% (*n* = 33) of the primary studies that were meta-analysed were conducted in high income countries. However, approximately 75% of suicides occur in low- and middle-income countries (LMIC), where rates of poverty are higher, and there are limited resources to support people experiencing suicidality [101]. In addition, there is a shortage of screening and diagnostic instruments for ASCs, along with a reduced awareness of autism in healthcare professions [102, 103]. The combination of these factors presents unique systemic challenges to autistic people in LMICs compared to higher income countries and limits the generalisability of our findings to all autistic populations.

The current review also only included samples of autistic and possibly autistic people without co-occurring ID, as this population was identified as higher risk [15]. However, autistic people with co-occurring ID are not exempt from suicidality; co-occurring ID in autistic people is found to be associated with an increased risk of suicide attempts/ self-injurious behaviour, but not suicidal ideation [37]. It may be that this finding reflects high levels of self-injurious behaviour in those with co-occurring ID [104] without necessarily having suicidal intent [28]. Alternatively, it could indicate difficulties in assessment of suicidal ideation in those with co-occurring ID where self-report measures present additional challenges for understanding and responding to questions, over and above those associated with being autistic [40]. This could lead to lower reports of internally experienced outcomes (i.e. suicidal ideation) but not outwardly observable behaviours (i.e. suicide attempts/ self-injurious behaviour). Future meta-analyses should aim to compare evidence of suicidality in autistic people with and without co-occurring ID to determine if this is the case.

The results of the review were also somewhat limited by the quality of primary studies and their methodology, where few demonstrated low risk of bias. To address this limitation, moderation analyses were carried out using risk of bias rating and total NOS score, but quality did not significantly influence prevalence rates across studies for any of the outcomes. Regardless, this still highlights the need for research in the field to better address sources of bias.

Finally, even though heterogeneity of suicidality measures were explored with subgroup analyses, there were still inherent differences in the ways that “suicidality” was conceptualised, making it difficult to draw concrete conclusions [105]. Studies generally did not distinguish passive suicidal ideation (i.e. desire to be dead) from active suicidal ideation (i.e. desire to kill oneself), and some used definitions of suicidal ideation that included suicide plans, while others consider suicide plans to be a discrete stage [106]. There were also wide variations in the observation period within which the measured suicidality outcomes occur, particularly for suicidal ideation (e.g. current, 6 months, 12 months, lifetime, etc.). This review also utilised a dichotomous conceptualisation of self-harm [107, 108]; however, not all literature distinguishes suicide attempts from self-harm. For example, some studies were excluded for using items encompassing both suicide attempts and self-injurious behaviour [25, 26]. It is therefore possible that some relevant literature may have been missed. Not only should future research aim to measure suicidality in autistic and possibly autistic people homogenously with validated measures (which is more feasible now using the SBQ-ASC [24]), but also provide clear and fine-grained categorisations of suicidality.

### Implications

Nonetheless, the high prevalence of suicidality in autistic and possibly autistic people found in the current review has important implications for suicide prevention both clinically and scientifically. Future research should continue to address priorities for better suicide prevention that are in line with those identified by the autistic community [109]. One such example is to adapt and develop methods that accurately measure relevant constructs (i.e. suicidality and self-harm) in autistic populations. It is essential that this process also be guided by recommendations from a validated research tool, such as the Consensus-Based Standards for the Selection of Health Measurement Instruments, which emphasises the importance of content validity [110].

Likewise, significant gaps in the literature as to *why* autistic people are more at risk of suicidality need to be addressed. While there is an overlap with known risk markers in the general population, these tend to be significantly more prevalent in autistic people, and others have been identified that are unique to autism [41]. Research should also explore whether such risk markers of suicidality extend to possibly autistic people too.

With an increased understanding of the epidemiology of suicidality in autistic and possibly autistic people, further research is also needed to explore the mechanisms underpinning both the development of suicidal ideation, and the progression from suicidal ideation to suicide attempts and behaviours [111]. This should be routed in theory such as the Interpersonal Theory of Suicide (IPTS) [112]. Theories of suicide have been underused in the autism field so far, but the IPTS has emerging utility within autistic and possibly autistic populations [75, 113]. The IPTS stipulates a combination of perceived burdensomeness and thwarted belongingness create a desire for suicide, and acquired capability to attempt suicide is dependent on overcoming fear of death and the pain that accompanies a suicide attempt [10]. Autistic people are more likely to report experiencing thwarted belongingness and perceived burdensomeness than non-autistic people, where both mediate the association between autistic traits and suicidality [76]. Likewise, in individuals with high autistic traits, camouflaging is associated with increased thwarted belongingness [114]. The IPTS could facilitate better understanding of suicidality in autistic and possibly autistic people by determining who is at risk of suicide, and therefore how to reduce this [40].

## Concluding remarks

In summary, the current meta-analysis has generated robust prevalence estimates for suicidal ideation, suicide plans and suicide attempts and behaviours in both autistic and possibly autistic people without co-occurring ID. Significant heterogeneity was found across primary studies, where moderator analysis demonstrated prevalence varied as a result of participant and study level characteristics. Prevalence estimates of suicidal ideation were lower for studies conducted in Asia, but higher in transgender or gender non-conforming samples and when using self-report. Prevalence estimates of suicide plans were higher for autistic adults and when using suicidality specific measures. Gender was also associated with suicide plans, where a decrease in the proportion of males was associated with an increase in estimates of suicide plans. Conversely, no variables were found to moderate prevalence of suicide attempts and behaviours. More research is needed, in partnership with the autistic community, to understand why the increased risk of suicidality exists in this population. Recommendations include better quality measures, evidence for risk or protective factors, and extension of theoretical models. This will aid suicide prevention by ensuring autistic and possibly autistic people experiencing suicidality receive the appropriate and timely support they need.

## Supplementary Information

**Additional file 1: Supplementary Materials 1.** Search Syntax and Terms for Each Database; **Supplementary Materials 2.** Adapted Newcastle–Ottawa Scale (NOS) Rating Scale; **Supplementary Materials 3.** Deviations from the PRISMA protocol; **Supplementary Materials 4.** Supplemental Reference List of Included Primary Studies; **Supplementary Materials 5.** Baujat Diagnostic Plot of Sources of Heterogeneity; **Supplementary Materials 6.** Funnel Plots of Prevalence Outcomes.

## Data Availability

The data that support the findings of this study are available from the corresponding author (VN) on reasonable request.

## Abbreviations

AQ: Autism quotient
ASC: Autism spectrum condition
DSM: Diagnostic and statistical manual of mental disorders
ICD: International classification of diseases
ID: Intellectual disability
IPTS: Interpersonal theory of suicide
LMIC: Low- and middle-income counties
NOS: Newcastle–Ottawa scale
PABAK: Prevalence- and bias-adjusted kappa
PRISMA: Preferred reporting for items for systematic reviews and meta-analysis
SBQ-ASC: Suicidal behaviours questionnaire-autism spectrum conditions

## Footnotes

1 Identity first language will be used to refer to individuals diagnosed with ASC (e.g. autistic community/ person/ individual), which is the preferred language of the autistic community [1].
