# Supplementary material for: A systematic review and meta-analysis of suicidality in autistic and possibly autistic people without co-occurring intellectual disability
Source: Mol Autism. 2023 Mar 15;14:12. doi: 10.1186/s13229-023-00544-7 (PMC10018918; doi:10.1186/s13229-023-00544-7)
Supplement: Supplementary file 1 — Additional file 1: Supplementary Materials 1. Search Syntax and Terms for Each Database; Supplementary Materials 2. Adapted Newcastle–Ottawa Scale (NOS) Rating Scale; Supplementary Materials 3. Deviations from the PRISMA protocol; Supplementary Materials 4. Supplemental Reference List of Included Primary Studies; Supplementary Materials 5. Baujat Diagnostic Plot of Sources of Heterogeneity; Supplementary Materials 6. Funnel Plots of Prevalence Outcomes. [file 13229_2023_544_MOESM1_ESM.docx]

**Additional File 1**

**Supplementary Materials 1: Search Syntax and Terms for Each Database**

**OVID** (APA PsycArticles full text**;** Embase 1980 to 2021; Ovid MEDLINE(R) ALL 1946 to January 2022; APA PsycINFO 1806 to January 2022)

1. (ASC or ASD or Asperg* or Autis* or 'high#functioning' or 'pervasive developmental disorder' or PDD or HFA).ti,ab.
2. ('possib* autis*' or 'autis* trait*' or 'autis* phenotyp*' or 'undiagnosed autis*' or 'self-diagnos* autis*').ti,ab.
3. (suicid* or 'suicide plans' or 'suicide attempts' or 'attempted suicide' or parasuicide 'self-harm' or 'self-inj*').ti,ab.
4. #1 or #2
5. #3 and #4
6. Limit #5 to yr=”1992 – current”

Autistic disorder [MeSH]

autism spectrum disorder [MeSH]

pervasive developmental disorder [MeSH]

suicidal ideation [MeSH]

suicidal behavior [MeSH]

attempted suicide [MeSH]

suicide [MeSH]

self-injurious behaviour [MeSH]

**PubMed**

1. ASC OR ASD OR Asperg* OR Autis* OR "high functioning" OR "pervasive developmental disorder" OR PDD OR HFA[Title/Abstract]
2. possibly autistic OR "autistic traits" OR "autism phenotype" OR "undiagnosed autis*" OR self-diagnos* autism[Title/Abstract]
3. suicid* OR parasuicide OR "suicide plans" OR "suicide attempts" OR "attempted suicide" OR "self-harm" OR "self-injur*"[Title/Abstract]
4. (((ASC OR ASD OR Asperg* OR Autis* OR "high functioning" OR "pervasive developmental disorder" OR PDD OR HFA[Title/Abstract]) OR ((possibly autistic OR "autistic traits" OR "autism phenotype" OR "undiagnosed autis*" OR self-diagnos* autism[Title/Abstract]))) AND (suicid* OR parasuicide OR "suicide plans" OR "suicide attempts" OR "attempted suicide" OR "self-harm" OR "self-injur*"[Title/Abstract])) AND (("1992"[Date - Publication] : "3000"[Date - Publication]))

**Web of Science**

**Refine:** English**,** 1992-2022

1. TI= (ASC OR ASD OR Asperg* OR Autis* OR "high functioning" OR "pervasive developmental disorder" OR PDD or HFA) AND AB=(ASC OR ASD OR Asperg* OR Autis* OR "high functioning" OR "pervasive developmental disorder" OR PDD OR HFA)
2. TI= ("possibl* autis*" OR "autis* trait*" OR "autis* phenotyp*" OR "undiagnosed autis*" OR "self-diagnos* autis*") AND AB=("possibl* autis*" OR "autis* trait*" OR "autis* phenotyp*" OR "undiagnosed autis*" OR "self-diagnos* autis*")
3. TI= (suicid* OR parasuicide OR "suicide plans" OR "suicide attempts" OR "attempted suicide" OR "self-harm" OR "self-inj*") AND AB= (suicid* OR parasuicide OR "suicide plans" OR "suicide attempts" OR "attempted suicide" OR "self-harm" OR "self-inj*")
4. #1 or #2
5. #3 and #4

**Supplementary Materials 2: Adapted Newcastle-Ottawa Scale (NOS) Rating Scale**

**Cross-sectional**

**Selection (maximum 5)**

1. Representativeness of the sample
   1. truly representative of the average in the autistic population **
      1. random sampling or whole population (e.g. national/ clinical/ birth registry)
      2. AT LEAST 25% or more females
   2. somewhat representative of the average in the autistic population *
      1. Non-random sampling (e.g. purposive sample, evidence sample is representative of source population)
      2. AT LEAST 25% or more females
   3. potential for selection biases
      1. selected group of users or convenience sample (e.g. students from one university, specific clinical sample) *OR* less than 25% females
   4. no description of the sampling strategy
2. Sample size
   1. justified and satisfactory (including sample size calculation) *
   2. sample size provided but not justified
   3. no description
      1. In cases of very large samples (e.g. n > 1000) it may be classified as (a)
3. Ascertainment of autism/ possible autism
   1. Validated diagnostic assessments (e.g. using versions of ADOS/ ADI) used to confirm autism diagnosis, administered by a trained professional in present study **
      1. *IF* possibly autistic group/sample – score above threshold on a validated screening tool for non-diagnostic purposes (e.g. >26 on versions of the AQ) **
   2. Previous formal diagnosis of autism is extracted from medical records/documented proof but unconfirmed in present study, or formal diagnosis of autism specified alongside a validated screening tool for non-diagnostic purposes/other methods of assessment *
   3. Self-reported diagnosis/suspected diagnosis with no additional evidence
   4. No description

**Comparability (maximum 1)**

1. Non-respondents
   1. comparability between respondents and non-respondents’ characteristics was established with a satisfactory response rate *
   2. the response rate was unsatisfactory, the comparability between respondents and non-respondents was unsatisfactory (i.e. no comparison made), or there was no description of the response rate or the characteristics of the responders or non-responders

**Outcome (maximum 3)**

1. Assessment of suicidality
   1. structured interview or self/informant-report using a validated measure of suicidality (e.g., versions of the SBQ-R, SIQ), *OR* record linkage (e.g. identified through ICD codes on database records) **
   2. validated measure NOT specific to suicidality but includes specific items or modules (e.g. ASRI, MOODS-SR, CBCL), *OR* information extracted from medical records *
   3. unstandardised questions about suicidality (e.g. yes/no, items with no reference to a specific measure)
   4. no description or unspecified (e.g. “a survey”)
2. Quality of descriptive statistics reporting
   1. Study reported descriptive statistics to describe the population (e.g. age, sex) with proper measures of dispersion (e.g. mean, standard deviation) *
   2. The study did not report descriptive statistics, incompletely reported descriptive statistics, or did not report measures of dispersion for descriptive statistics

Low risk of bias: 7-9

Unclear risk of bias: 4-6

High risk of bias: 0-3

**Cohort**

**Selection (maximum 4)**

1. Representativeness of the sample
   1. truly representative of the average in the autistic population *
      1. random sampling or whole population (e.g. national/ clinical/ birth registry)
      2. AT LEAST 25% or more females
   2. somewhat representative of the average in the autistic population *
      1. Non-random sampling (e.g. purposive sample, evidence sample is representative of source population)
      2. AT LEAST 25% or more females
   3. potential for selection biases
      1. selected group of users or convenience sample (e.g. students from one university, specific clinical sample) *OR* less than 25% females
   4. no description of the sampling strategy
2. Selection of the non-exposed cohort
   1. drawn from the same community as the exposed cohort *
   2. drawn from a different source
   3. no description of the derivation of the non-exposed cohort
3. Ascertainment of autism/ possible autism
   1. Validated diagnostic assessments (e.g. using versions of ADOS/ ADI) used to confirm autism diagnosis, administered by a trained professional in present study *
      1. *IF* possibly autistic group/sample – score above threshold on a validated screening tool for non-diagnostic purposes (e.g. >26 on versions of the AQ) *
   2. Previous formal diagnosis of autism is extracted from medical records/documented proof but unconfirmed in present study, or formal diagnosis of autism specified alongside a validated screening tool for non-diagnostic purposes/other methods of assessment *
   3. Self-reported diagnosis/suspected diagnosis with no additional evidence
   4. No description
4. Demonstration that suicidality was accounted for or not present at start of study
   1. yes *
   2. no

**Comparability (maximum 2)**

1. Comparability of cohorts on the bases of the design or analysis
   1. Study controls for co-occurring mental health conditions. Either exposed and non-exposed individuals must be matched in the design and/or confounders be adjusted for in the analysis
      1. Statements of no differences between groups or that differences were not statistically significant are not sufficient for establishing comparability *
   2. Study controls for additional demographic characteristic (e.g., sex/ gender, age) *
   3. Both **
   4. None

**Outcome (maximum 3)**

1. Assessment of suicidality
   1. structured interview or self/informant-report using a validated measure of suicidality (e.g., versions of the SBQ-R, SIQ), *OR* record linkage (e.g. identified through ICD codes on database records) *
   2. validated measure NOT specific to suicidality but includes specific items or modules (e.g. ASRI, MOODS-SR, CBCL), *OR* information extracted from medical records *
   3. unstandardised questions about suicidality (e.g. yes/no, items with no reference to a specific measure)
   4. no description or unspecified (e.g. “a survey”)
2. Was follow-up long enough for outcomes to occur?
   1. yes (< 1 year) *OR* retrospective *
   2. no
3. Adequacy of follow-up of cohorts
   1. complete follow up - all subjects accounted for *
   2. subjects lost to follow but rate given (description provided of those lost) *
   3. follow up rate not adequate (> 20%) or no description of subjects lost to follow up
   4. no statement

Low risk of bias: 7-9

Unclear risk of bias: 4-6

High risk of bias: 0-3

**Case-control**

**Selection (maximum 4)**

1. Is the case definition adequate?
   1. yes, with independent validation (e.g. >1 person/record/assessment/process to extract information, or reference to primary medical records) *
   2. yes, based on informant or self-report
   3. no description
2. Representativeness of the cases
   1. consecutive or obviously representative series of cases *
   2. potential for selection biases or not stated
3. Selection of controls
   1. community controls from source population (i.e. same community as cases and would be cases if had outcome) *
   2. clinical controls if clinical source population *
   3. not extracted from same source population
   4. no description
4. Definition of controls
   1. no history of outcome evident at endpoint
      1. If cases have new (not necessarily first) occurrence of outcome, controls with previous occurrences should not be excluded) *
   2. no mention of history or outcome

**Comparability (maximum 2)**

1. Comparability of cohorts on the bases of the design or analysis
   1. Study controls for co-occurring mental health conditions. Either cases and controls must be matched in the design and/or confounders be adjusted for in the analysis
      1. Statements of no differences between groups or that differences were not statistically significant are not sufficient for establishing comparability. *
   2. Study controls for additional demographic characteristic (e.g., sex/ gender, age *
   3. Both **
   4. None

**Exposure (maximum 3)**

1. Ascertainment of exposure
   1. Validated measure or diagnostic tool *
   2. Medical records or record linkage *
   3. Unstandardised self or informant report only
   4. No description
2. Same method of exposure ascertainment for cases and controls
   1. yes *
   2. no
3. Non-respondents
   1. comparability between respondents and non-respondents’ characteristics was established with a satisfactory response rate (>60%) *
   2. the response rate was unsatisfactory, the comparability between respondents and non-respondents was unsatisfactory (i.e. no comparison made), or there was no description of the response rate or the characteristics of the responders or non-responders.

Low risk of bias: 7-9

Unclear risk of bias: 4-6

High risk of bias: 0-3

**Supplemental Materials 3: Deviations from PROSPERO Protocol**

1. The PROSPERO protocol described the condition or domain being studied as “suicidal ideation/ thoughts or behaviours (suicide plans or attempts/parasuicides) and death by suicide”. However, based on the data available, we did not have enough estimates of deaths by suicide for it to be a focus point in the current review. Alternatively, we found that enough studies gave estimates for suicide plans, which was explored as a suicidality outcome instead.

**Supplementary Materials 4: Supplemental Reference List of Included Primary Studies**

Anderson, A. H., Carter, M., & Stephenson, J. (2020). An On-Line Survey of University Students with Autism Spectrum Disorder in Australia and New Zealand: Characteristics, Support Satisfaction, and Advocacy. *J Autism Dev Disord, 50*(2), 440-454. doi:10.1007/s10803-019-04259-8

Arwert, T. G., & Sizoo, B. B. (2020). Self-reported Suicidality in Male and Female Adults with Autism Spectrum Disorders: Rumination and Self-esteem. *J Autism Dev Disord, 50*(10), 3598-3605. doi:10.1007/s10803-020-04372-z

Bal, V. H., Leventhal, B. L., Carter, G., Kim, H., Koh, Y. J., Ha, M., . . . Kim, Y. S. (2022). Parent-Reported Suicidal Ideation in Three Population-Based Samples of School-Aged Korean Children With Autism Spectrum Disorder and Autism Spectrum Screening Questionnaire Screen Positivity. *Arch Suicide Res, 26*(3), 1232-1249. doi:10.1080/13811118.2020.1868367

Balfe, M., & Tantam, D. (2010). A descriptive social and health profile of a community sample of adults and adolescents with Asperger syndrome. *BMC research notes, 3*(1), 1-7

Cassidy, S., Bradley, L., Shaw, R., & Baron-Cohen, S. (2018). Risk markers for suicidality in autistic adults. *Mol Autism, 9*, 42. doi:10.1186/s13229-018-0226-4

Cassidy, S., Bradley, P., Robinson, J., Allison, C., McHugh, M., & Baron-Cohen, S. (2014). Suicidal ideation and suicide plans or attempts in adults with Asperger's syndrome attending a specialist diagnostic clinic: a clinical cohort study. *The Lancet Psychiatry, 1*(2), 142-147. doi:10.1016/s2215-0366(14)70248-2

Cassidy, S. A., Bradley, L., Cogger-Ward, H., & Rodgers, J. (2021). Development and validation of the suicidal behaviours questionnaire - autism spectrum conditions in a community sample of autistic, possibly autistic and non-autistic adults. *Molecular Autism, 12*(1), 46. doi:http://dx.doi.org/10.1186/s13229-021-00449-3

Chang, J. C., Lai, M. C., Tai, Y. M., & Gau, S. S. (2022). Mental health correlates and potential childhood predictors for the wish to be of the opposite sex in young autistic adults. *Autism, 26*(1), 146-159. doi:10.1177/13623613211024098

Chaplin, E., McCarthy, J., Underwood, L., Allely, C. S., Forrester, A., Hayward, H., . . . Mills, R. (2021). Self-harm and Mental Health Characteristics of Prisoners with elevated rates of autistic traits. *Research in Developmental Disabilities, 114*, 103987. doi:http://dx.doi.org/10.1016/j.ridd.2021.103987

Costa, A. P., Loor, C., & Steffgen, G. (2020). Suicidality in Adults with Autism Spectrum Disorder: The Role of Depressive Symptomatology, Alexithymia, and Antidepressants. *J Autism Dev Disord, 50*(10), 3585-3597. doi:10.1007/s10803-020-04433-3

Dell'Osso, L., Carpita, B., Muti, D., Morelli, V., Salarpi, G., Salerni, A., . . . Maj, M. (2019). Mood symptoms and suicidality across the autism spectrum. *Compr Psychiatry, 91*, 34-38. doi:10.1016/j.comppsych.2019.03.004

Dow, D., Morgan, L., Hooker, J. L., Michaels, M. S., Joiner, T. E., Woods, J., & Wetherby, A. M. (2021). Anxiety, Depression, and the Interpersonal Theory of Suicide in a Community Sample of Adults with Autism Spectrum Disorder. *Arch Suicide Res, 25*(2), 297-314. doi:10.1080/13811118.2019.1678537

Green, J., Gilchrist, A., Burton, D., & Cox, A. (2000). Social and psychiatric functioning in adolescents with Asperger syndrome compared with conduct disorder. *Journal of autism and developmental disorders, 30*(4), 279-293.

Greger, H. K., Myhre, A. K., Lydersen, S., & Jozefiak, T. (2015). Previous maltreatment and present mental health in a high-risk adolescent population. *Child Abuse Negl, 45*, 122-134. doi:10.1016/j.chiabu.2015.05.003

Hedley, D., Uljarevic, M., Foley, K. R., Richdale, A., & Trollor, J. (2018). Risk and protective factors underlying depression and suicidal ideation in Autism Spectrum Disorder. *Depress Anxiety, 35*(7), 648-657. doi:10.1002/da.22759

Hirvikoski, T., Boman, M., Chen, Q., D'Onofrio, B. M., Mittendorfer-Rutz, E., Lichtenstein, P., . . . Larsson, H. (2020). Individual risk and familial liability for suicide attempt and suicide in autism: a population-based study. *Psychol Med, 50*(9), 1463-1474. doi:10.1017/S0033291719001405

Hooijer, A. A. T., & Sizoo, B. B. (2020). Temperament and character as risk factor for suicide ideation and attempts in adults with autism spectrum disorders. *Autism Res, 13*(1), 104-111. doi:10.1002/aur.2221

Hu, H. F., Liu, T. L., Hsiao, R. C., Ni, H. C., Liang, S. H., Lin, C. F., . . . Yen, C. F. (2019). Cyberbullying Victimization and Perpetration in Adolescents with High-Functioning Autism Spectrum Disorder: Correlations with Depression, Anxiety, and Suicidality. *J Autism Dev Disord, 49*(10), 4170-4180. doi:10.1007/s10803-019-04060-7

Jackson, S. L. J., Hart, L., Brown, J. T., & Volkmar, F. R. (2018). Brief Report: Self-Reported Academic, Social, and Mental Health Experiences of Post-Secondary Students with Autism Spectrum Disorder. *J Autism Dev Disord, 48*(3), 643-650. doi:10.1007/s10803-017-3315-x

Karakoc Demirkaya, S., Tutkunkardas, M. D., & Mukaddes, N. M. (2016). Assessment of suicidality in children and adolescents with diagnosis of high functioning autism spectrum disorder in a Turkish clinical sample. *Neuropsychiatr Dis Treat, 12*, 2921-2926. doi:10.2147/NDT.S118304

Kim, D.-Y., Maisashvili, T., Khawaja, A., Song, A., Strang, J. F., Willing, L., . . . Vilain, E. (2021). In Addition to Stigma: Cognitive and Autism-Related Predictors of Mental Health in Transgender Adolescents. *Journal of clinical child and adolescent psychology : the official journal for the Society of Clinical Child and Adolescent Psychology, American Psychological Association, Division 53*, 1-18. doi:http://dx.doi.org/10.1080/15374416.2021.1916940

Moseley, R. L., Gregory, N. J., Smith, P., Allison, C., & Baron-Cohen, S. (2020). Links between self-injury and suicidality in autism. *Mol Autism, 11*(1), 14. doi:10.1186/s13229-020-0319-8

Moses, T. (2017). Suicide Attempts Among Adolescents with Self-Reported Disabilities. *Child Psychiatry & Human Development*. doi:10.1007/s10578-017-0761-9

Mukaddes, N. M., & Fateh, R. (2010). High rates of psychiatric co-morbidity in individuals with Asperger's disorder. *The World Journal of Biological Psychiatry, 11*(2-2), 486-492.

Paquette-Smith, M., Weiss, J., & Lunsky, Y. (2014). History of suicide attempts in adults with Asperger syndrome. *Crisis, 35*(4), 273-277. doi:10.1027/0227-5910/a000263

Pelton, M. K., Crawford, H., Robertson, A. E., Rodgers, J., Baron-Cohen, S., & Cassidy, S. (2020). Understanding Suicide Risk in Autistic Adults: Comparing the Interpersonal Theory of Suicide in Autistic and Non-autistic Samples. *J Autism Dev Disord, 50*(10), 3620-3637. doi:10.1007/s10803-020-04393-8

Pilunthanakul, T., Goh, T. J., Fung, D. S. S., Sultana, R., Allen, J. C., & Sung, M. (2021). Validity of the patient health questionnaire 9-item in autistic youths: a pilot study. *BMC Psychiatry, 21*(1), 564. doi:10.1186/s12888-021-03556-w

Raja, M., Azzoni, A., & Frustaci, A. (2011). Autism spectrum disorders and suicidality. *Clinical practice and epidemiology in mental health: CP & EMH, 7*, 97.

Sharpley, C. F., Bitsika, V., Andronicos, N. M., & Agnew, L. L. (2016). Further evidence of HPA-axis dysregulation and its correlation with depression in Autism Spectrum Disorders: Data from girls. *Physiol Behav, 167*, 110-117. doi:10.1016/j.physbeh.2016.09.003

Shtayermman, O. (2008). Suicidal Ideation and Comorbid Disorders in Adolescents and Young Adults Diagnosed with Asperger's Syndrome: A Population at Risk. *Journal of Human Behavior in the Social Environment, 18*(3), 301-328. doi:10.1080/10911350802427548

Shtayermman, O. (2019). A Brief Report on Suicidal Ideation in Adolescents Diagnosed with Asperger Syndrome. *Advances in Neurodevelopmental Disorders, 4*(1), 46-50. doi:10.1007/s41252-019-00142-w

South, M., Beck, J. S., Lundwall, R., Christensen, M., Cutrer, E. A., Gabrielsen, T. P., . . . Lundwall, R. A. (2020). Unrelenting Depression and Suicidality in Women with Autistic Traits. *J Autism Dev Disord, 50*(10), 3606-3619. doi:10.1007/s10803-019-04324-2

Storch, E. A., Sulkowski, M. L., Nadeau, J., Lewin, A. B., Arnold, E. B., Mutch, P. J., . . . Murphy, T. K. (2013). The phenomenology and clinical correlates of suicidal thoughts and behaviors in youth with autism spectrum disorders. *J Autism Dev Disord, 43*(10), 2450-2459. doi:10.1007/s10803-013-1795-x

Strauss, P., Cook, A., Watson, V., Winter, S., Whitehouse, A., Albrecht, N., . . . Lin, A. (2021). Mental health difficulties among trans and gender diverse young people with an autism spectrum disorder (ASD): Findings from Trans Pathways. *J Psychiatr Res, 137*, 360-367. doi:10.1016/j.jpsychires.2021.03.005

Takara, K., & Kondo, T. (2014). Autism spectrum disorder among first-visit depressed adult patients: diagnostic clues from backgrounds and past history. *Gen Hosp Psychiatry, 36*(6), 737-742. doi:10.1016/j.genhosppsych.2014.08.004

Takara, K., & Kondo, T. (2014). Comorbid atypical autistic traits as a potential risk factor for suicide attempts among adult depressed patients: a case–control study. *Annals of general psychiatry, 13*(1), 1-8

Umeda, M., Ishikawa, H., Kawakami, N., Shimoda, H., Miyamoto, K., Tachimori, H., & Takeshima, T. (2021). Comorbidity and sociodemographic characteristics of adult autism spectrum disorder and attention deficit hyperactivity disorder: epidemiological investigation in the World Mental Health Japan 2nd Survey. *International Journal of Developmental Disabilities, 67*(1), 58-66. doi:http://dx.doi.org/10.1080/20473869.2019.1576409

Weibel, S., Coutelle, R., Bemmouna, D., & Weiner, L. (2021). Feasibility, Acceptability and Preliminary Efficacy of Dialectical Behavior Therapy for Autistic Adults without Intellectual Disability: A Mixed Methods Study. *Journal of Autism and Developmental Disorders*. doi:http://dx.doi.org/10.1007/s10803-021-05317-w

Wijnhoven, L. A., Niels-Kessels, H., Creemers, D. H., Vermulst, A. A., Otten, R., & Engels, R. C. (2019). Prevalence of comorbid depressive symptoms and suicidal ideation in children with autism spectrum disorder and elevated anxiety symptoms. *J Child Adolesc Ment Health, 31*(1), 77-84. doi:10.2989/17280583.2019.1608830

Zhou, N., Wang, J., & Chasson, G. S. (2018). Psychiatric problems of Chinese college students with high autism traits. *Research in Autism Spectrum Disorders, 54*, 1-8. doi:10.1016/j.rasd.2018.06.008

**Supplementary Materials 5: Baujat Diagnostic Plot of Sources of Heterogeneity**

*Note. The vertical axis reports the influence of the study on the overall effect and the horizontal axis reports the discrepancy of the study with the rest of the literature.*

**Figure 1**

**
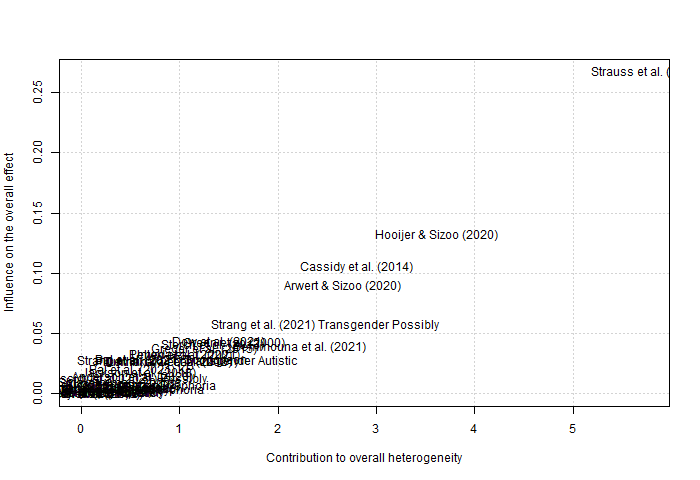
**Suicidal Ideation

**Influential analysis (Random effects model)**

PR 95%-CI p-value tau^2 tau I^2

Omitting Anderson et al. (2020) 0.3379 [0.2738; 0.4020] < 0.0001 0.0336 0.1832 95.8%

Omitting Arwert & Sizoo (2020) 0.3324 [0.2704; 0.3944] < 0.0001 0.0311 0.1765 95.7%

Omitting Bal et al. (2021) CHEER 0.3442 [0.2795; 0.4089] < 0.0001 0.0342 0.1848 95.9%

Omitting Bal et al. (2021) KP 0.3465 [0.2823; 0.4108] < 0.0001 0.0336 0.1834 95.6%

Omitting Bal et al. (2021) Sooncheon 0.3474 [0.2835; 0.4113] < 0.0001 0.0333 0.1824 95.8%

Omitting Balfe & Tantam (2010) 0.3403 [0.2759; 0.4048] < 0.0001 0.0340 0.1845 95.9%

Omitting Bemmouna et al. (2021) 0.3355 [0.2729; 0.3982] < 0.0001 0.0323 0.1798 95.9%

Omitting Cassidy et al. (2014) 0.3316 [0.2699; 0.3934] < 0.0001 0.0308 0.1754 94.3%

Omitting Cassidy et al. (2021) Autistic 0.3440 [0.2793; 0.4087] < 0.0001 0.0342 0.1849 95.9%

Omitting Cassidy et al. (2021) Possibly 0.3382 [0.2740; 0.4024] < 0.0001 0.0337 0.1835 95.8%

Omitting Chang et al. (2021) Gender Dysphoria 0.3392 [0.2751; 0.4032] < 0.0001 0.0337 0.1836 95.9%

Omitting Chang et al. (2021) No Gender Dsyphoria 0.3438 [0.2793; 0.4084] < 0.0001 0.0341 0.1848 95.9%

Omitting Chaplin et al. (2021) Autistic 0.3440 [0.2799; 0.4081] < 0.0001 0.0338 0.1840 95.9%

Omitting Chaplin et al. (2021) Possibly 0.3430 [0.2785; 0.4076] < 0.0001 0.0341 0.1847 95.9%

Omitting Demirkaya et al. (2016) 0.3472 [0.2833; 0.4111] < 0.0001 0.0333 0.1825 95.9%

Omitting Dow et al. (2021) 0.3486 [0.2851; 0.4120] < 0.0001 0.0327 0.1809 95.8%

Omitting Green et al. (2000) 0.3486 [0.2853; 0.4118] < 0.0001 0.0326 0.1807 95.9%

Omitting Greger et al. (2015) 0.3482 [0.2846; 0.4118] < 0.0001 0.0329 0.1814 95.8%

Omitting Hooijer & Sizoo (2020) 0.3306 [0.2695; 0.3916] < 0.0001 0.0300 0.1732 95.6%

Omitting Jackson et al. (2018) 0.3462 [0.2821; 0.4104] < 0.0001 0.0336 0.1834 95.9%

Omitting Moseley et al (2020) 0.3435 [0.2789; 0.4082] < 0.0001 0.0342 0.1849 95.9%

Omitting Pelton et al. (2020) 0.3477 [0.2839; 0.4116] < 0.0001 0.0332 0.1821 95.7%

Omitting Pilunthanakul et al. (2021) 0.3472 [0.2833; 0.4112] < 0.0001 0.0333 0.1826 95.8%

Omitting Raja et al. (2011) 0.3427 [0.2784; 0.4070] < 0.0001 0.0340 0.1845 95.9%

Omitting Sharpley et al. (2016) 0.3416 [0.2771; 0.4061] < 0.0001 0.0341 0.1848 95.9%

Omitting Shtayermman (2008) 0.3391 [0.2754; 0.4028] < 0.0001 0.0335 0.1830 95.9%

Omitting Shtayermman (2020) 0.3450 [0.2809; 0.4090] < 0.0001 0.0337 0.1837 95.9%

Omitting South et al. (2019) Autistic 0.3410 [0.2766; 0.4053] < 0.0001 0.0340 0.1844 95.9%

Omitting South et al. (2019) Possibly 0.3406 [0.2761; 0.4050] < 0.0001 0.0341 0.1846 95.9%

Omitting Storch et al. (2013) 0.3484 [0.2849; 0.4119] < 0.0001 0.0328 0.1811 95.8%

Omitting Strang et al. (2021) Cisgender 0.3442 [0.2798; 0.4086] < 0.0001 0.0340 0.1843 95.9%

Omitting Strang et al. (2021) Transgender Autistic 0.3365 [0.2731; 0.4000] < 0.0001 0.0330 0.1816 95.9%

Omitting Strang et al. (2021) Transgender Possibly 0.3343 [0.2718; 0.3967] < 0.0001 0.0319 0.1786 95.8%

Omitting Strauss et al. (2021) 0.3265 [0.2681; 0.3849] < 0.0001 0.0271 0.1646 94.0%

Omitting Umeda et al. (2021) 0.3478 [0.2840; 0.4116] < 0.0001 0.0331 0.1820 95.8%

Omitting Wijnhoven et al. (2019) 0.3423 [0.2777; 0.4070] < 0.0001 0.0342 0.1851 95.9%

Omitting Zhou et al. (2018) 0.3430 [0.2785; 0.4075] < 0.0001 0.0341 0.1848 95.9%

Pooled estimate 0.3419 [0.2790; 0.4047] < 0.0001 0.0331 0.1821 95.8%

After omitting one study showing a disproportionate influence (Strauss et al., 2021), the corrected random-effects model reported a pooled prevalence estimate for SI as 0.3265 (z= 10.96, p < .001; 95% CI: 0.2681 – 0.3849) This model evidences an approximately 4.5% decrease relative to the uncorrected estimate but did not alter the overall meta-analytic effect.

**Figure 2**


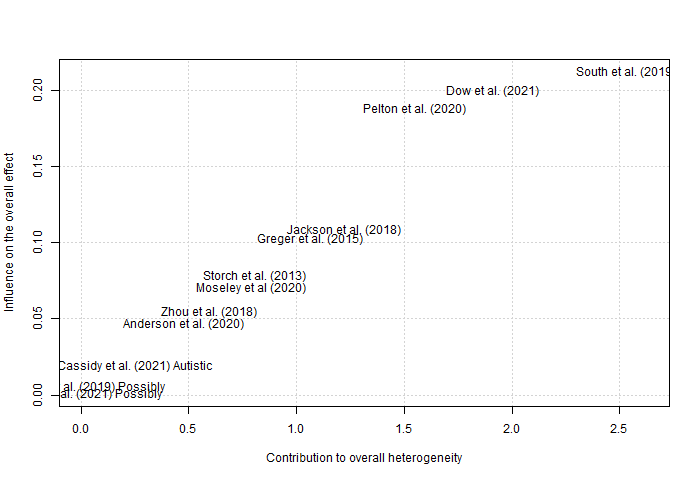
Suicide Plans

**Influential analysis (Random effects model)**

PR 95%-CI p-value tau^2 tau I^2

Omitting Anderson et al. (2020) 0.2292 [0.1378; 0.3205] < 0.0001 0.0217 0.1474 96.2%

Omitting Cassidy et al. (2021) Autistic 0.2126 [0.1204; 0.3048] < 0.0001 0.0221 0.1487 95.7%

Omitting Cassidy et al. (2021) Possibly 0.2205 [0.1271; 0.3140] < 0.0001 0.0228 0.1511 96.2%

Omitting Dow et al. (2021) 0.2382 [0.1544; 0.3219] < 0.0001 0.0178 0.1335 92.9%

Omitting Greger et al. (2015) 0.2336 [0.1451; 0.3221] < 0.0001 0.0202 0.1423 96.1%

Omitting Jackson et al. (2018) 0.2045 [0.1181; 0.2910] < 0.0001 0.0195 0.1397 96.0%

Omitting Moseley et al (2020) 0.2071 [0.1182; 0.2959] < 0.0001 0.0205 0.1433 95.9%

Omitting Pelton et al. (2020) 0.2005 [0.1161; 0.2848] < 0.0001 0.0182 0.1348 93.8%

Omitting South et al. (2019) Autistic 0.2000 [0.1186; 0.2814] < 0.0001 0.0174 0.1319 96.0%

Omitting South et al. (2019) Possibly 0.2226 [0.1298; 0.3153] < 0.0001 0.0226 0.1504 96.2%

Omitting Storch et al. (2013) 0.2320 [0.1421; 0.3218] < 0.0001 0.0209 0.1446 96.2%

Omitting Zhou et al. (2018) 0.2299 [0.1393; 0.3205] < 0.0001 0.0214 0.1464 96.2%

Pooled estimate 0.2191 [0.1342; 0.3040] < 0.0001 0.0204 0.1429 95.9%

After omitting one autistic group (South et al., 2019) and additional two studies which showed disproportionate influence (Dow et al., 2021; Pelton et al., 2020). The corrected random-effects model reported a pooled prevalence estimate for SP as 0.1969 (z= 5.04, p < .001; 95% CI: 0.1204 – 0.2734). This model evidences an approximately 10.1% decrease relative to the uncorrected estimate but did not alter the overall meta-analytic effect

**Figure 3**

**
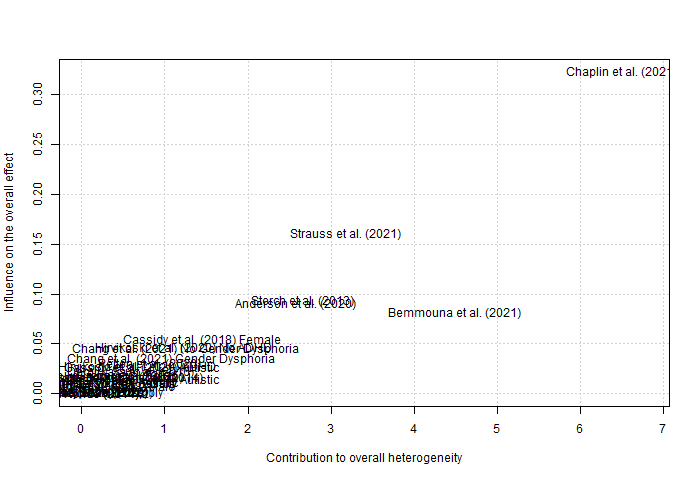
**Suicide Attempts and Behaviours

**Influential analysis (Random effects model)**

PR 95%-CI p-value tau^2 tau I^2

Omitting Anderson et al. (2020) 0.2508 [0.1979; 0.3037] < 0.0001 0.0188 0.1372 96.6%

Omitting Arwert & Sizoo (2020) 0.2413 [0.1859; 0.2967] < 0.0001 0.0210 0.1449 96.7%

Omitting Balfe & Tantam (2010) 0.2462 [0.1910; 0.3014] < 0.0001 0.0208 0.1443 96.8%

Omitting Bemmouna et al. (2021) 0.2350 [0.1827; 0.2874] < 0.0001 0.0189 0.1374 96.7%

Omitting Cassidy et al. (2018) Female 0.2362 [0.1824; 0.2901] < 0.0001 0.0197 0.1404 96.6%

Omitting Cassidy et al. (2018) Male 0.2402 [0.1851; 0.2954] < 0.0001 0.0208 0.1443 96.7%

Omitting Cassidy et al. (2021) Autistic 0.2381 [0.1834; 0.2929] < 0.0001 0.0204 0.1428 96.4%

Omitting Cassidy et al. (2021) Possibly 0.2435 [0.1878; 0.2991] < 0.0001 0.0212 0.1455 96.8%

Omitting Chang et al. (2021) Gender Dysphoria 0.2479 [0.1934; 0.3024] < 0.0001 0.0203 0.1425 96.8%

Omitting Chang et al. (2021) No Gender Dysphoria 0.2485 [0.1941; 0.3030] < 0.0001 0.0201 0.1418 96.8%

Omitting Chaplin et al. (2021) Autistic 0.2394 [0.1852; 0.2936] < 0.0001 0.0203 0.1426 96.8%

Omitting Chaplin et al. (2021) Possibly 0.2285 [0.1797; 0.2773] < 0.0001 0.0159 0.1260 96.6%

Omitting Demirkaya et al. (2016) 0.2468 [0.1918; 0.3019] < 0.0001 0.0207 0.1439 96.8%

Omitting Dow et al. (2021) 0.2447 [0.1892; 0.3003] < 0.0001 0.0211 0.1453 96.8%

Omitting Greger et al. (2015) 0.2389 [0.1840; 0.2937] < 0.0001 0.0205 0.1432 96.7%

Omitting Hirvikoski et al. (2020) ADHD 0.2473 [0.1922; 0.3024] < 0.0001 0.0207 0.1437 96.0%

Omitting Hirvikoski et al. (2020) No ADHD 0.2487 [0.1942; 0.3031] < 0.0001 0.0201 0.1418 95.8%

Omitting Hooijer & Sizoo (2020) 0.2416 [0.1861; 0.2970] < 0.0001 0.0210 0.1450 96.7%

Omitting Jackson et al. (2018) 0.2463 [0.1911; 0.3015] < 0.0001 0.0208 0.1443 96.8%

Omitting Moseley et al (2020) 0.2415 [0.1860; 0.2970] < 0.0001 0.0211 0.1451 96.7%

Omitting Moses (2018) 0.2452 [0.1896; 0.3008] < 0.0001 0.0211 0.1452 96.8%

Omitting Mukaddes & Fateh (2010) 0.2455 [0.1903; 0.3008] < 0.0001 0.0209 0.1446 96.8%

Omitting Paquette-Smith et al. (2014) 0.2391 [0.1843; 0.2939] < 0.0001 0.0205 0.1433 96.7%

Omitting Pelton et al. (2020) 0.2378 [0.1831; 0.2924] < 0.0001 0.0203 0.1425 96.3%

Omitting Pilunthanakul et al. (2021) 0.2435 [0.1879; 0.2992] < 0.0001 0.0212 0.1455 96.8%

Omitting South et al. (2019) Autistic 0.2456 [0.1905; 0.3008] < 0.0001 0.0209 0.1444 96.8%

Omitting South et al. (2019) Possibly 0.2455 [0.1901; 0.3008] < 0.0001 0.0210 0.1448 96.8%

Omitting Storch et al. (2013) 0.2509 [0.1981; 0.3037] < 0.0001 0.0187 0.1369 96.4%

Omitting Strauss et al. (2021) 0.2321 [0.1804; 0.2838] < 0.0001 0.0179 0.1338 96.3%

Omitting Takara & Kondo (2014) 0.2430 [0.1874; 0.2986] < 0.0001 0.0211 0.1454 96.8%

Omitting Takara & Kondo (2014) 0.2430 [0.1876; 0.2983] < 0.0001 0.0211 0.1451 96.8%

Pooled estimate 0.2426 [0.1890; 0.2963] < 0.0001 0.0203 0.1424 96.7%

After omitting the autistic group from one study which showed a disproportionate influence (Chaplin et al., 2021). The corrected random-effects model reported a synthesis of suicide attempts and behaviours as 0.2394 (z= 8.66, p < .001; 95% CI: 0.1852 – 0.2936) This model evidences an approximately 1.3% decrease relative to the uncorrected estimate but did not alter the overall meta-analytic effect.

**Supplementary Materials 6: Funnel plots of prevalence outcomes.**

The 95% confidence interval of the expected distribution of prevalence is shown as an inverted “funnel”.

**Figure 1**


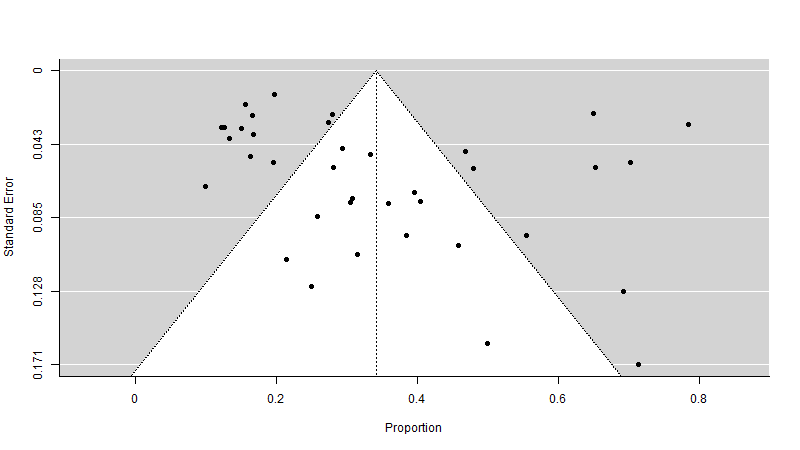
Suicidal Ideation

**Linear regression test of funnel plot asymmetry**

Test result: t = 1.60, df = 35, p-value = 0.1192

Sample estimates:

bias se.bias intercept se.intercept

2.3069 1.4444 0.2084 0.0563

Details:

- multiplicative residual heterogeneity variance (tau^2 = 22.7096)

- predictor: standard error

- weight: inverse variance

- reference: Egger et al. (1997), BMJ

**Figure 2**


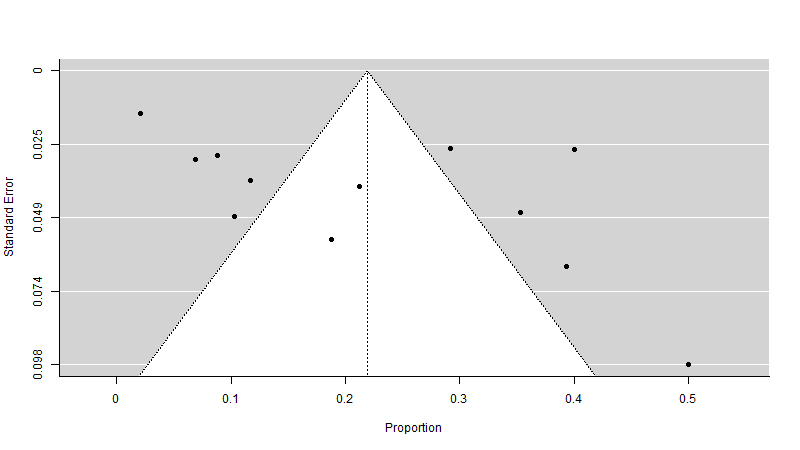
Suicide Plans

**Linear regression test of funnel plot asymmetry**

Test result: t = 2.05, df = 10, p-value = 0.0671

Sample estimates:

bias se.bias intercept se.intercept

5.6328 2.7434 -0.0040 0.0827

Details:

- multiplicative residual heterogeneity variance (tau^2 = 18.6938)

- predictor: standard error

- weight: inverse variance

- reference: Egger et al. (1997), BMJ

**Figure 3**

**
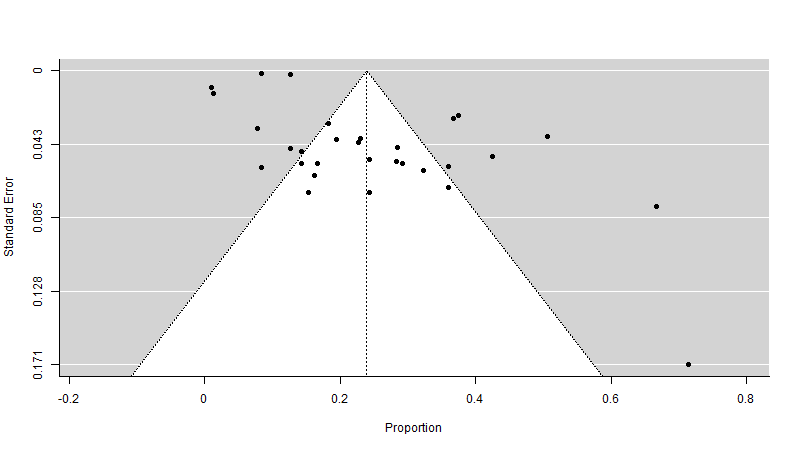
**Suicide Attempts and Behaviours.

**Linear regression test of funnel plot asymmetry**

Test result: t = 3.27, df = 28, p-value = 0.0028

Sample estimates:

bias se.bias intercept se.intercept

3.1909 0.9756 0.0898 0.0074

Details:

- multiplicative residual heterogeneity variance (tau^2 = 23.2113)

- predictor: standard error

- weight: inverse variance

- reference: Egger et al. (1997), BMJ

**Trim and fill correction**

Estimated number of missing studies on the left side: 0 (SE = 3.4716)

Random-Effects Model (k = 34; tau^2 estimator: REML)

tau^2 (estimated amount of total heterogeneity): 0.0205 (SE = 0.0057)

tau (square root of estimated tau^2 value): 0.1433

I^2 (total heterogeneity / total variability): 99.44%

H^2 (total variability / sampling variability): 178.49

Test for Heterogeneity:

Q(df = 33) = 990.3162, p-val < .0001

Model Results:

estimate se zval pval ci.lb ci.ub

0.2306 0.0263 8.7840 <.0001 0.1792 0.2821 ***
